# Ovariectomy Impaired Hepatic Glucose and Lipid Homeostasis and Altered the Gut Microbiota in Mice With Different Diets

**DOI:** 10.3389/fendo.2021.708838

**Published:** 2021-06-30

**Authors:** Zili Lei, Huijuan Wu, Yanhong Yang, Qing Hu, Yuting Lei, Wanwan Liu, Ya Nie, Lanxiang Yang, Xueying Zhang, Changyuan Yang, Ting Lin, Fengxue Tong, Jiamin Zhu, Jiao Guo

**Affiliations:** ^1^ Guangdong Metabolic Diseases Research Center of Integrated Chinese and Western Medicine, Key Laboratory of Glucolipid Metabolic Disorder, Ministry of Education of China, Institute of Chinese Medicine, Guangdong Traditional Chinese Medicine (TCM) Key Laboratory for Metabolic Diseases, Guangdong Pharmaceutical University, Guangzhou, China; ^2^ School of Traditional Chinese Medicine, Guangdong Pharmaceutical University, Guangzhou Higher Education Mega Center, Guangzhou, China; ^3^ The First Affiliated Hospital (School of Clinical Medicine), Guangdong Pharmaceutical University, Guangzhou, China

**Keywords:** ovariectomy, glucose and lipid metabolism, glycogen storage, lipid accumulation, gut microbiota

## Abstract

The lower incidence of metabolic diseases of women than men and the increasing morbidity of metabolic disorders of menopausal women indicated that hormones produced by ovaries may affect homeostasis of glucose and lipid metabolism, but the underlying mechanisms remain unclear. To explore the functions of ovaries on regulating glucose and lipid metabolism in females, 8 weeks old C57BL/6 mice were preformed ovariectomy and administrated with normal food diet (NFD) or high fat diet (HFD). Six weeks after ovariectomy, blood biochemical indexes were tested and the morphology and histology of livers were checked. The expression levels of genes related to glucose and lipid metabolism in liver were detected through transcriptome analysis, qPCR and western blot assays. 16S rDNA sequence was conducted to analyze the gut microbiota of mice with ovariectomy and different diets. The serum total cholesterol (TC) was significantly increased in ovariectomized (OVX) mice fed with NFD (OVXN), and serum low density lipoprotein-cholesterol (LDL-C) was significantly increased in both OVXN mice and OVX mice fed with HFD (OVXH). The excessive glycogen storage was found in livers of 37.5% mice from OVXN group, and lipid accumulation was detected in livers of the other 62.5% OVXN mice. The OVXN group was further divided into OVXN-Gly and OVXN-TG subgroups depending on histological results of the liver. Lipid drops in livers of OVXH mice were more and larger than other groups. The expression level of genes related with lipogenesis was significantly increased and the expression level of genes related with β-oxidation was significantly downregulated in the liver of OVXN mice. Ovariectomy also caused the dysbiosis of intestinal flora of OVXN and OVXH mice. These results demonstrated that hormones generated by ovaries played important roles in regulating hepatic glucose and lipid metabolism and communicating with the gut microbiota in females.

## Introduction

The prevalence of glucose and lipid metabolic disorders is increasing worldwide (1, 2) and metabolic diseases are more prevalent in men than in age-matched women (3–5). Female hormones have been considered to play important roles in regulating glucose homeostasis and lipid metabolism in humans and mice (6). It has been reported that estradiol (E2) increased insulin secretion in humans (7). E2 also could protect pancreatic β-cells through improving oxidative stress, lipotoxicity, amyloid polypeptide toxicity and apoptosis in rodent models of type1 and type 2 diabetes mellitus (8). Several studies have confirmed that estrogen deficiency would increase metabolic dysfunctions, including obesity, type 2 diabetes and the metabolic syndrome, even certain cancers (9–11). Estrogen is generated by the ovaries and it has been reported that lower E2 levels caused by ovaries aging were related to 47% higher risk of developing diabetes in women during the early menopausal transition (12). Recent study demonstrated that cardiovascular disease (CVD) became the leading cause of death among women of United States because of early menopause and diminished ovarian reserve (13). Towfighi et al. (14) reported that the prevalence of myocardial infarction (MI) was increasing among midlife women. It has been confirmed that menopause was one of risk factors for glucose and lipid metabolic disorders, including type 2 diabetes, metabolic syndrome and CDV (15). Nowadays, the elderly population was increasing faster than the total population all over the world (16), and it would be very important to pay attention to the health of elderly women. Ovariectomized rodents are very useful animal models to study geriatric diseases. For example, ovariectomy is a frequently used technique to induce osteoporosis in rodent models (17–19). Rodrigues et al. (20) found that physical exercise could alter the morphology of the liver of ovariectomized Ldlr^-/-^ mice. It was also reported that the lemon balm extract could improve the NAFLD and obesity in ovariectomized mice (21). Ovariectomized mice showed impaired glucose tolerance and elevated body fat, which could not be mitigated by exercise training (22). Kim et al. (21) also reported the obesity and nonalcoholic fatty liver disease (NALFD) phenotype in ovariectomized female mice. These studies indicated that ovaries had important functions in maintaining the homeostasis of glucose and lipid metabolism, and it would be very meaningful to elucidate the molecular mechanisms of ovaries on regulating metabolism in females.

The liver was essential to regulate glucose and lipid metabolism (23, 24), and the pathological state of liver was related with the abnormality of metabolism (25). Several studies have demonstrated that ectopic accumulation of lipid within liver could specifically cause hepatic insulin resistance in humans and rodents (26–29). Jackson et al. (30) found the increase of the monounsaturated fatty acid composition of hepatic triglyceride in the liver of ovariectomized mice. Oliveira et al. (31) also reported that the liver of the long-term estrogen deficiency of ovariectomized mice showed considerable accretions of the lipid contents. In human beings, it seemed that the prevalence of NAFLD was higher in postmenopausal women than in premenopausal women, and NAFLD added extra morbidity to postmenopausal women, possibly *via* increasing the risk of T2DM and cardiovascular disease (32). Recently, Quinn et al. (33) reported that estrogen deficiency could cause NAFLD through a glucocorticoid receptor (GR) signaling pathway independent of hepatic ERα, but they had not introduced the function of estrogen on hepatocytes through ERα yet. However, more than 1,000 genes showed the sex-bias in their expression in human liver (34), which indicated that estrogen might have important functions in regulating the expression of genes in liver. ChIP assay results confirmed that at least 43 genes related with lipid metabolism were transcriptionally regulated by ERα in the liver of mice (35). These studies have revealed that ovaries played important roles in regulating glucose and lipid metabolism in liver, but the underlying mechanisms of hepatic metabolic disorders with menopause in females still needs further study.

Gut microbiota was one of the important regulators of host metabolism, and host and gut microbiota could interact with each other (36). However, few studies focused on the interactions between ovaries and gut microbiota. Fuhrman et al. (37) reported that the diversity and composition of the fecal microbiota were associated with the urinary estrogens and estrogen metabolites in postmenopausal women, and women with elevated urinary ratio of hydroxylated estrogen metabolites to parent estrogen showed a more diverse gut microbiome. Cox-York et al. (38) found that the number of the *Bacteroidetes* phylum and microbial diversity in ovariectomized rats of low intrinsic aerobic capacity were significantly increased compared with the Sham rats. Using ovariectomized mice, Choi et al. (39) found that the gut microbiome in mice of menopausal obesity was similar to that of obesity mice induced by high fat diet (HFD), but still having its own specific bacteria. These works have demonstrated that there were multiple interactions between ovaries and gut microbiota, and the molecular mechanism of these interactions needs to be explored.

In the present study, the molecular mechanisms of ovaries on regulating the hepatic glucose and lipid metabolism and interacting with gut microbiota were studied by using ovariectomized mice administrated with normal food diet (NFD) or HFD, providing new insights about the relationship between female hormones and metabolism related diseases.

## Materials and Methods

### Mice

**Figure 1:**
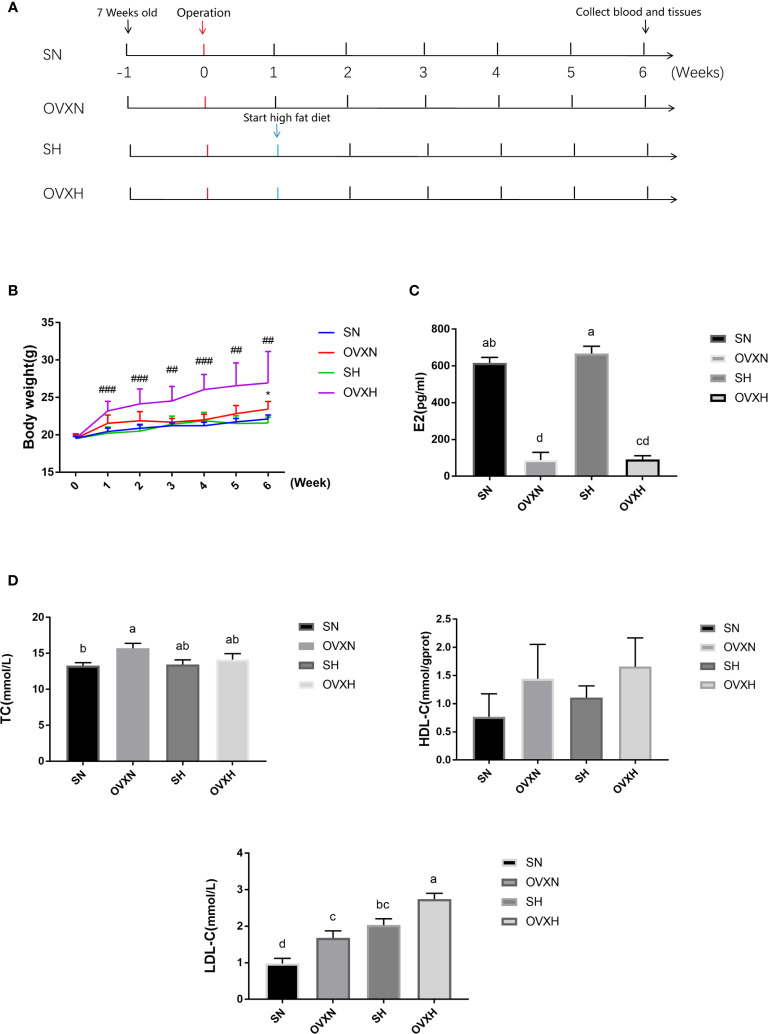
The effect of ovariectomy on the body weight and blood biochemical indexes of mice with NFD or HFD. **(A)** The overview of the experimental design. **(B)** The body weight curves within 6weeks after operation of mice from the SN, OVXN, SH and OVXH groups. **(C, D)** The concentration of estradiol (E2), TC, HDL-C, and LDL-C in the serum of mice from the SN, OVXN, SH and OVXH groups at six weeks after operation. One-way ANOVA was used to compare the difference between two groups; and ^*^P < 0.05 compared with the SN group, ^##^P < 0.01, ^###^P < 0.001 compared with the SH group. Two-way ANOVA was performed to compare the difference among more than two groups; a-d, meaning significant difference among groups. SN, sham operated control mice fed with normal food diet; OVXN, ovariectomized mice fed with normal food diet; SH, sham operated control mice fed with high fat diet; OVXH, ovariectomized mice fed with high fat diet.

All animal experimental procedures were reviewed and approved by the Committee of Laboratory Animal Care and Use of Guangdong Pharmaceutical University (Guangzhou, China). A total of 60 female C57BL/6 mice (7 weeks old) purchased from Hunan Lex Jingda Laboratory Animal Co., Ltd. (Changsha, Hunan Province, China), were housed in a SPF (specific pathogen-free) animal facility, 60-65% humidity, 12hr light-dark cycle, at 25°C, with free access to water and food. After one week of acclimatization, 49 mice were selected and divided into 4 groups according to the body weight, with the similar average body weight of each group. After that, two groups of mice were undergone ovariectomy and another two control groups were treated with sham operation. One week after operation, one group of ovariectomized mice were administrated with HFD (Research Diets, Inc., D12492, 26.2% protein, 26.3% carbohydrate, 34.9% fat) (OVXH group, n=13), another group of ovariectomized mice were continued to be treated with NFD (BEIJING KEAO XILI FEED CO., LTD., 2212, 23.07% protein, 65.08% carbohydrate, 11.85% fat) (OVXN group, n=11). The sham operated control mice were also divided into 2 groups, treated with NFD (SN group, n=12) and HFD (SH group, n=13) respectively (**Figure 1A**). The body weight of the mice was measured once a week. After 6 weeks of operation, the blood and liver tissues were collected for biochemical, histological and transcriptional tests; and stool samples were harvested in the last week of the experimental process and were frozen in −80˚C. The mice which were used in the present study had been summarized in **Table S1**.

### Assays of the Blood Biochemical Profile

The concentrations of estradiol (E2), total cholesterol (TC), triglyceride (TG), high density lipoprotein-cholesterol (HDL-C), low density lipoprotein-cholesterol (LDL-C), alanine aminotransferase (ALT), aspartate aminotransferase (AST) in serum, and fasting plasm glucose (FPG) were measured according to the manufacturer’s protocols for each kit. The determination kit for E2 (CSB-E05109m, Wuhan, China) was purchased from CUSABIO BIOTECH CO. Ltd, and determination kits for TC (A111-1-1, Nanjing, China), TG (A110-1-1, Nanjing, China), HDL-C (A112-1-1, Nanjing, China), LDL-C (A113-1-1, Nanjing, China), ALT (C009-2-1, Nanjing, China), AST (C010-2-1, Nanjing, China), and FPG (F006-1-1, Nanjing, China) were purchased from Nanjing Jiancheng Bioengineering Institute.

### Histological Staining

Mouse liver tissues were fixed in 4% paraformaldehyde at 4°C overnight for histological staining. For hematoxylin and eosin (H&E) staining, 4-µm-thick paraffin sections were stained with hematoxylin (H9627-100G, Sigma-Aldrich) for 3 min and followed with eosin (E4009-25G, Sigma-Aldrich) for 20 sec at room temperature. For oil-red O staining, 7-µm-thick frozen sections were stained with oil-red O (O0625, Sigma-Aldrich) for 10 min at room temperature. The periodic acid Schiff (PAS) kit (G1281, Solarbio, Beijing, China) was used for PAS staining, 4-µm-thick paraffin sections were stained with periodic acid for 8 min, then Schiff regent for 15min, and followed with hematoxylin (H9627-100G) for 3min at room temperature. The images were captured with a PerkinElmer Automated Quantitative Pathology System (PerkinElmer, Inc.).

### Transcriptome Analysis

Liver tissues of mice were quickly frozen in liquid nitrogen and then stored at −80°C. RNA extraction, library construction, sequencing, and the transcriptome analysis were conducted by Gene Denovo Biotechnology Company (Guangzhou, China). The procedures were simply described here. Total RNA of each tissue was extracted using Trizol reagent kit (15596-018, Invitrogen), and the RNA quality was assessed through an Agilent 2100 Bioanalyzer (Agilent Technologies) and then checked using RNase free agarose gel electrophoresis. After the extraction, eukaryotic mRNAs were enriched *via* Oligo (dT) beads. Enriched mRNAs were fragmented in the fragmentation buffer, and then were reverse transcribed into cDNA using random primers. The cDNA fragments were purified *via* AMPure XP beads (A63880, Beckman), subsequently end repaired, poly (A) added, and then ligated to Illumina sequencing adapters. The ligation products were size separated through agarose gel electrophoresis, then PCR amplified, and finally sequenced *via* Illumina HiSeq2500. Reads were filtered through fastp (version 0.18.0). Differential expression analysis was performed *via* DESeq2 software. Differentially expressed mRNAs were considered with the parameter of false discovery rate (FDR) less than 0.05 and the absolute fold change≥2.

To perform Gene Ontology (GO) analysis on differentially expressed genes (DEGs), all DEGs were mapped to GO terms (the basic unit of GO) in the Gene Ontology database (http://www.geneontology.org/), gene numbers were calculated for every term, significantly enriched GO terms in DEGs comparing to the genome background were defined by hypergeometric test. The calculated p-value were gone through FDR Correction, taking FDR ≤ 0.05 as a threshold. GO terms meeting this condition were defined as significantly enriched GO terms in DEGs.

Kyoto Encyclopedia of Genes and Genomes (KEGG) is the major public pathway-related database to analyze identified significantly enriched metabolic pathways or signal transduction pathways in DEGs comparing with the whole genome background. The calculating formula is the same as that in GO analysis. The calculated p-value was gone through FDR Correction, taking FDR ≤ 0.05 as a threshold. Pathways meeting this condition were defined as significantly enriched pathways in DEGs.

### Quantitative Reverse Transcription-Polymerase Chain Reaction

Total RNA was extracted from each liver tissue of mice by Trizol reagent (T9108, Takara Bio, Inc.), and then was subjected to reverse transcription through the PrimeScript™ RT Reagent kit (RR047A, Takara Bio, Inc.) at 37°C for 15 min and then 85°C for 5 sec. qPCR was performed using the SYBR Premix Ex Taq kit (RR820A, Takara Bio, Inc.) with the LightCycler 480II System (Roche, Inc.). The processes of cycling were: 95°C for 30 sec; and then followed by 40 cycles of 95°C for 5 sec, 60°C for 20 sec and 65°C for 15 sec. GAPDH was used as internal reference. All primers used for qPCR were listed in **Table S2**.

### Western Blot

Liver tissues were lysed in Radio-Immunoprecipitation Assay lysis buffer (MA0151, Dalian Meilun Biotechnology co., Ltd., Dalian, China), and then centrifuged at 13,680 x g, 4°C, 30 min, and then the supernatant was harvested. Protein concentration was measured *via* BCA kit (P0011, Beyotime, Shanghai, China). After that, equal amounts of protein (30 μg) were separate *via* the SDS-PAGE, and subsequently transferred to the PVDF membrane. The PVDF membrane was blocked with 5% skimmed milk (0040895, Biosharp, Hefei, China) in TBST buffer for 1 hour at room temperature, then incubated with primary antibodies in 4°C for overnight, and then incubated with HRP (horseradish peroxidase)-labeled secondary antibodies, the signals were detected using enhanced chemiluminescence reagent. The primary antibodies included: rabbit anti-GAPDH (1:2,500; ab9485; Abcam), rabbit anti-CD36 (1:1000; ab133625; Abcam), mouse anti-HNF4a (1:5,000; ab41898; Abcam), rabbit anti-HMGCR (1:1,000; ab174830; Abcam), mouse anti-SCD1 (1:1,000; ab19862; Abcam), rabbit anti-FASN (1:1,000; #3180; CST). Secondary antibodies included HRP-goat anti-rabbit IgG (1:5,000; os0701; Earthox Life Sciences) and HRP-donkey anti-mouse IgG (1:2,000; ab150105; Abcam). The quantification of WB bands was analyzed through the Lane 1d software (version 5.1.0.0; SageCreation).

### 16S rDNA Gene Analysis

Fecal samples of mice were quickly frozen in liquid nitrogen and then stored at −80°C. Fecal bacterial DNA extraction, 16S rDNA gene PCR amplification, sequencing, and analysis were performed by Gene Denovo Biotechnology Company (Guangzhou, China). The experimental procedures were conducted as previously introduced (40, 41). Fecal DNA was amplified *via* primers which flank the V3-V4 regions of bacterial 16S rDNA gene (42). The product of PCR amplification was harvested by gel cutting and quantified using Life Invitrogen Qubit 3.0 fluorometer. Then the purified PCR products were mixed in equal amount, added sequencing adaptors to construct the libraries. The raw tags were conducted under filtering conditions to get high-quality clean tags. Sequences were assigned to operational taxonomic units (OTUs) through the Uparse software (version 9.2.64_i86linux32; http://www.drive5.com/uparse) with a 97% threshold of pairwise identity. The OTU abundance information was normalized by the relative abundance which is to compute the OTU ratio in a sample or group to avoid the errors in the process of PCR and sequencing. The analysis was conducted at each taxonomical level (Phylum, Class, Order, Family, and Genus) separately. Subsequent analysis of function prediction, alpha diversity and beta diversity were all conducted based on the output normalized data.

The relative abundance was always used in differential taxa abundance analysis. Differential taxa abundance analysis between two groups was calculated by Welch’s t-test and Wilcoxon rank test in R package (version 2.5.3). Differential taxa abundance analysis among more than two groups was computed by Tukey’s HSD test and Kruskal.test in R package (version 2.5.3).

For the downstream analysis of 16S rDNA sequencing analysis, alpha diversity index was calculated in QIIME (version 1.9.1). OTU rarefaction curve were plotted in R project ggplot2 package (version 2.2.1). Alpha diversity index comparison between groups was calculated by Welch’s t-test and Wilcoxon rank test in R Project Vegan package (version 2.5.3). Alpha diversity index comparison among groups was computed by Tukey’s HSD test and Kruskal.test in R Project Vegan package (version 2.5.3).

For beta diversity analysis, sequence alignment was performed using Muscle (version 3.8.31) and phylogenetic tree was constructed using FastTree (version 2.1), then weighted and unweighted unifrac distance matrix were generated by GuniFrac package (version 1.0) in R project. Jaccard and bray-curtis distance matrix calculated in R Project Vegan package (version 2.5.3). PCA (principal component analysis) was performed in R Project Vegan package (version 2.5.3). Multivariate statistical techniques including PCoA (principal coordinates analysis) and NMDS (non-metric multi-dimensional scaling) of (Un) weighted unifrac, jaccard and bray-curtis distances were generated in R Project Vegan package (version 2.5.3) and plotted in R project ggplot2 package (version 2.2.1). Statistical analysis of Welch’s t-test, Wilcoxon rank test, Tukey’s HSD test, Kruskal.test, Adonis (also called Permanova) and Anosim test was calculated in R Project Vegan package (version 2.5.3).

For function prediction on microbial communities, the KEGG pathway analysis of the OTUs was inferred using Tax4Fun (version 1.0). Microbiome phenotypes of bacteria were classified using BugBase. FAPROTAX database (Functional Annotation of Prokaryotic Taxa) and associated software (version 1.0) were used for generating the ecological functional profiles of bacteria. Analysis of function difference between groups was calculated by Welch’s t-test, Wilcoxon rank test and Kruskal.test, Tukey’s HSD test in R Project Vegan package (version 2.5.3).

### Statistical Analysis

Statistical differences were determined by the SPSS software (version 25.0; IBM Corp.). Mean ± SE was used to express data. One-way ANOVA was conducted between two groups, and Two-way ANOVA was used when more than two groups were compared. P-value <0.05 was considered to be significant.

## Results

### Ovariectomy Increased Body Weight and Serum Concentration of LDL-C of Female Mice

To investigate the function of ovaries in regulating glucose and lipid metabolism in females, C57BL/6 female mice underwent ovariectomy at the age of 8 weeks (**Figure 1A**). The body weight of ovariectomized (OVX) mice was increased more quickly than that of the sham operated control mice; the body weight of mice from OVXH group started to be significantly higher than that of SH group after one week of operation, and the difference between OVXN and SN groups became statistically significant at 6 weeks after operation (**Figure 1B**), although there was no significant difference of the food intake among the mice of four groups (**Figure S1A**). The concentration of estradiol (E2) in the serum of ovariectomized mice was significantly reduced compared with the SN and SH groups (**Figure 1C**), which indicated that the ovariectomized operation was successful. The serum TC of the OVXN mice was significantly increased compared with the SN group (**Figure 1D**), and the serum LDL-C of both OVXN and OVXH groups was also significantly increased compared with the sham groups (**Figure 1D**). The concentration of serum LDL-C in the SH group was significantly higher than that of SN group, although the TC concentration in the serum was no significant difference between these two groups (**Figure 1D**); and the LDL-C concentration in the serum of OVXH group was also significantly increased compared with the OVXN group (**Figure 1D**). The concentration of the fasting plasm glucose (FPG) was increased in the mice of OVXN, SH, and OVXH groups, but not significant (**Figure S1B**). However, the concentration of ALT and AST in the serum of ovariectomized mice was not affected (**Figures S1D, E**). These results demonstrated that both ovariectomy and HFD could induce the increase of the serum LDL-C in females, however the increase of the body weight of female mice was only caused by ovariectomy within six weeks after operation.

### Ovariectomy Affected the Hepatic Glucose and Lipid Metabolism of Mice

**Figure 2:**
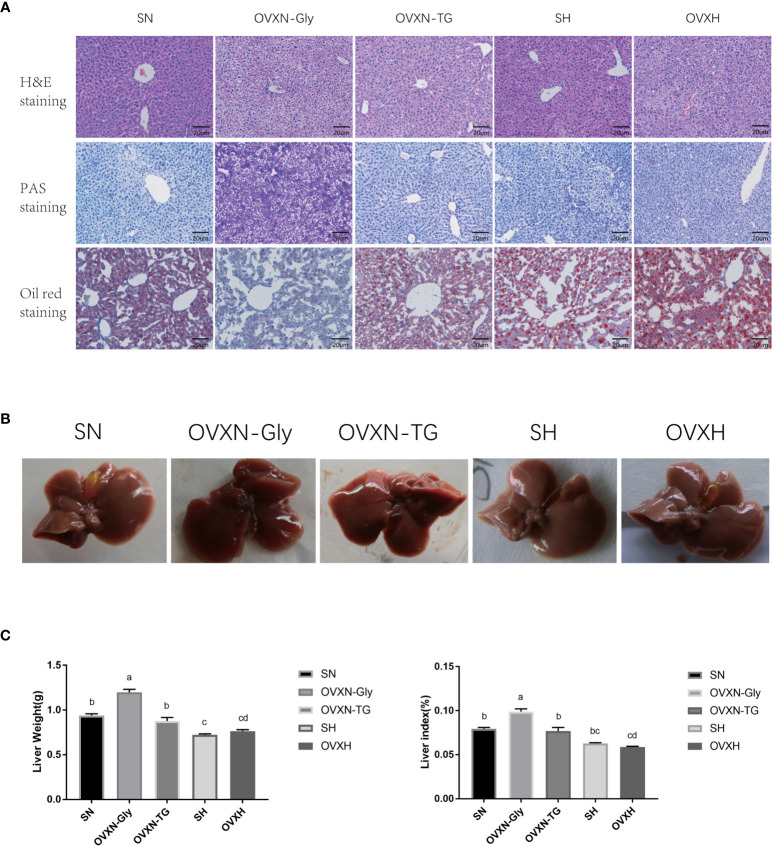
The histology and morphology of livers from ovariectomized and control mice with NFD or HFD. **(A)** Images of liver sections of H&E, PAS, and Oil Red O staining of mice from the SN, OVXN, SH and OVXH groups at six weeks after operation. **(B)** The morphology of livers of mice from the SN, OVXN, SH and OVXH groups after 6 weeks of operation. **(C)** Two-way ANOVA was performed to compare the weight and the weight index of the liver of mice from the SN, OVXN, SH and OVXH groups at six weeks after operation. a-d, meaning significant difference among groups. Scale bar, 50 μm. SN, sham operated control mice fed with normal food diet; OVXN-Gly, ovariectomized mice fed with normal food diet and glycogen accumulated in its liver; OVXN-TG, ovariectomized mice fed with normal food diet and triglyceride accumulated in its liver; SH, sham operated control mice fed with high fat diet; OVXH, ovariectomized mice fed with high fat diet.

Since the liver is an important organ to regulate the concentration of LDL-C in the blood (43, 44), the morphology and histology of the liver of ovariectomized mice were checked. The histology of the liver of mice from the SN, OVXN, SH and OVXH groups was tested *via* H&E staining, and the results showed that there were lipid drops accumulated in all the livers of mice from the SH and OVXH groups, but only 62.5% (5/8) of livers from mice of OVXN groups had lipid drops accumulation; moreover, the results of oil red O staining confirmed the accumulation of lipid drops in these livers, and the lipid drops in OVXH mice was more and larger than that of other groups (**Figure 2A**). The other 37.5% livers (3/8) of OVXN mice had increased cytoplasmic vacuolation, suggesting the increase of hepatic glycogen storage, and the sections of these livers showed dramatically increased PAS signal checked by PAS staining (**Figure 2A**), which was usually used to detect polysaccharides including glycogen (41, 45). These histological results demonstrated that the glucose and lipid metabolism was abnormal in the livers of ovariectomized mice and NAFLD is more easily to be induced by HFD in ovariectomized mice than in sham operated mice. According to the accumulation of glycogen or triglyceride (TG), the OVXN mice were further divided into the OVXN-Gly and OVXN-TG sub-groups (**Figure 2A**).

The morphology of livers from OVXN-TG mice was similar to that of the sham mice (**Figure 2B**). However, livers from OVXN-Gly group were larger than the SN group, and the liver weight and liver index of OVXN-Gly mice were significantly higher than that of the other three groups (**Figure 2C**). The liver weight and liver index of mice from OVXN-TG group were lower than that of OVXN-Gly group, but both the liver weight and the liver index of OVXN-TG mice were significantly higher than the OVXH group (**Figure 2C**).

### Ovariectomy Caused Abnormal Expression of Genes Related With Glucose And Lipid Metabolism in the Liver of Mice

In order to study the molecular mechanism of regulating the hepatic glucose and lipid metabolism by ovaries, the transcriptome analysis of the liver from the ovariectomized and control mice was performed (SRA: PRJNA699121), and differentially expressed genes were selected with the parameter of FDR< 0.05 and the absolute fold change≥2. There were 385 upregulated and 220 downregulated genes in the OVXN-Gly group compared with the SN group (**Figures S2A, B**). There were 131 upregulated and 84 downregulated genes in the OVXH group compared with the SH group (**Figures S2A, C**). There were 289 upregulated and 365 downregulated genes in OVXN-Gly group compared with the OVXN-TG group (**Figures S2A, D**). There were only 8 upregulated and 4 downregulated genes in the OVXN-TG group compared with the SN group (**Figures S2A, E**). The correlation of one of the samples in SN group with the other two samples was lower but still above 0.95 (**Figure S3A**), and there was no great separation of PCA between SN and OVXN-TG samples (**Figure S3B**); so the number of differentially expressed genes between SN and OVXN-TG was low. However, if the parameters for selection were set to P value<0.05 and the absolute fold change≥2, there were 155 upregulated and 49 downregulated genes in the OVXN-TG group compared with the SN group (**Figure S4**). Differently expressed genes of SN *vs* SH, OVXN-Gly *vs* OVXH, OVXN-TG *vs* OVXH groups were shown in **Figure S5A** and **Figures S6A, B**, respectively. Lists of differentially expressed genes for each comparison have been provided in **Tables S3**– **S10**.

**Figure 3:**
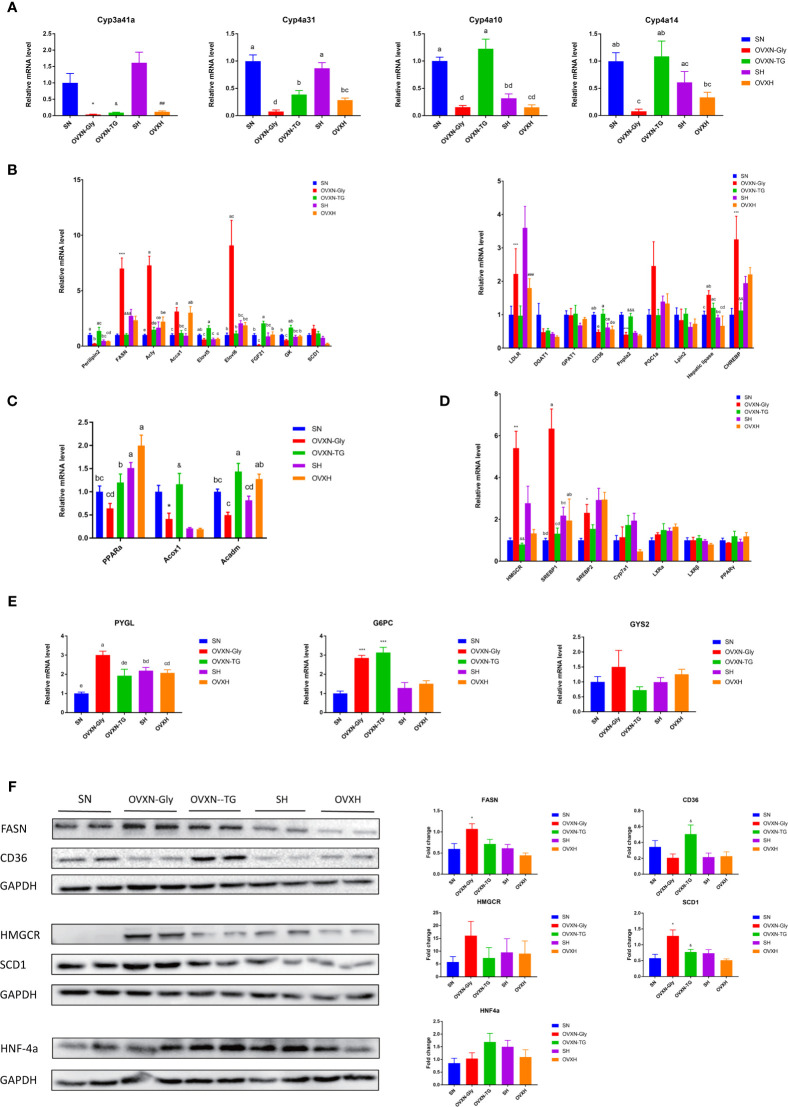
Testing differentially expressed genes in the transcriptomic profile of livers from ovariectomized and control mice with NFD or HFD *via* qPCR and Western blot. **(A)** Expression analysis of female-predominant genes, **(B)** genes related with lipogenesis, **(C)** PPARα and its target genes, **(D)** cholesterol and **(E)** glucose metabolism in the liver of mice from the SN, OVXN-Gly, OVXN-TG, SH and OVXH groups checked by qPCR. **(F)** The protein expression level analysis of FASN, CD36, HMGCR, SCD1 and PPARα in the liver of mice from the SN, OVXN-Gly, OVXN-TG, SH and OVXH groups checked by Western blot. One-way ANOVA was used to compare the difference between two group; and ^*^P < 0.05, ^**^P < 0.01, ^***^P < 0.001 compared with the SN group, ^##^P < 0.01, ^###^P < 0.001 compared with the SH group, ^&^P < 0.05, ^&&^P < 0.01, ^&&&^P < 0.001 compared with the OVXN-Gly group. Two-way ANOVA was performed to compare the difference among more than 2 groups; a-e, meaning significant difference among groups. SN, sham operated control mice fed with normal food diet; OVXN-Gly, ovariectomized mice fed with normal food diet and glycogen accumulated in its liver; OVXN-TG, ovariectomized mice fed with normal food diet and triglyceride accumulated in its liver; SH, sham operated control mice fed with high fat diet; OVXH, ovariectomized mice fed with high fat diet.

Results of qPCR assay confirmed that the expression of Cyp3a41a, which was specially expressed in liver of female mice (46), was significantly downregulated in the ovariectomized mice compared with the sham operated control mice (**Figure 3A**), and the mRNA level in the liver of SH group was significantly higher than the SN group (**Figure 3A**). Cyp4a10 and Cyp4a14, which were female-predominantly expressed in liver of mice (47), were significantly downregulated in the OVXN-Gly group compared with the SN group, but the expression of Cyp4a10 and Cyp4a14 were significantly upregulated in the OVXN-TG group compared with the OVXN-Gly group (**Figure 3A**). Many differentially expressed genes from transcriptome assay were related with glucose and lipid metabolism, and their relative expression levels in the liver were further checked using qPCR (**Figures 3A–E**). The expression of Perilipin2, which usually localized on the surface of lipid drops, was increased in the OVXN-TG group compared with the SN group but the increase was not significant (**Figure 3B**). However, it was significantly downregulated in the OVXN-Gly group compared with SN and OVXN-TG groups (**Figure 3B**). The expression of genes involved in fatty acid and triacylglycerol synthesis, such as Fasn, Acly, Acca1, Elovl6 and Chrebp, was significantly increased in the OVXN-Gly group compared with the SN group, while their expression levels were significantly downregulated in the OVXN-TG group compared with the OVXN-Gly group (**Figure 3B**). The expression levels of PPARα and its target gene Acadm were decreased in the OVXN-Gly group compared with SN group, but the reduction of them was not significant (**Figure 3C**). However, the expression levels of other PPARα target genes including Acox1, Cyp4a10, Cyp4a14 and Cyp4a31 were all significantly reduced in the OVXN-Gly group compared with SN mice (**Figures 3A, C**). These results indicated that the activity of PPARα might be affected in the liver of OVXN-Gly mice. The expression levels of genes related with cholesterol metabolism, such as HMGCR, SREBP1, and SREBP2, were also significantly increased in the liver of OVXN-Gly group compared with the SN group, but their expression levels were not significantly altered in the OVXN-TG group compared to SN mice (**Figure 3D**). The expression level of G6PC, which was related to glucose metabolism, was significantly upregulated both in the OVXN-Gly and the OVXN-TG groups compared with the SN group (**Figure 3E**). The mRNA level of PYGL was also significantly higher in the liver of OVNN-Gly mice than that of SN mice (**Figure 3E**). The alteration of the expression of these genes confirmed the results of transcriptome analysis, and the comparison of the fold-change in RNA-seq and qPCR validation analysis was listed in **Table S11**. The changes of the expression levels of FASN, CD36, HMGCR, SCD1 and HNF4α proteins were similar with their mRNA levels in the liver of mice (**Figure 3F**).

**Figure 4:**
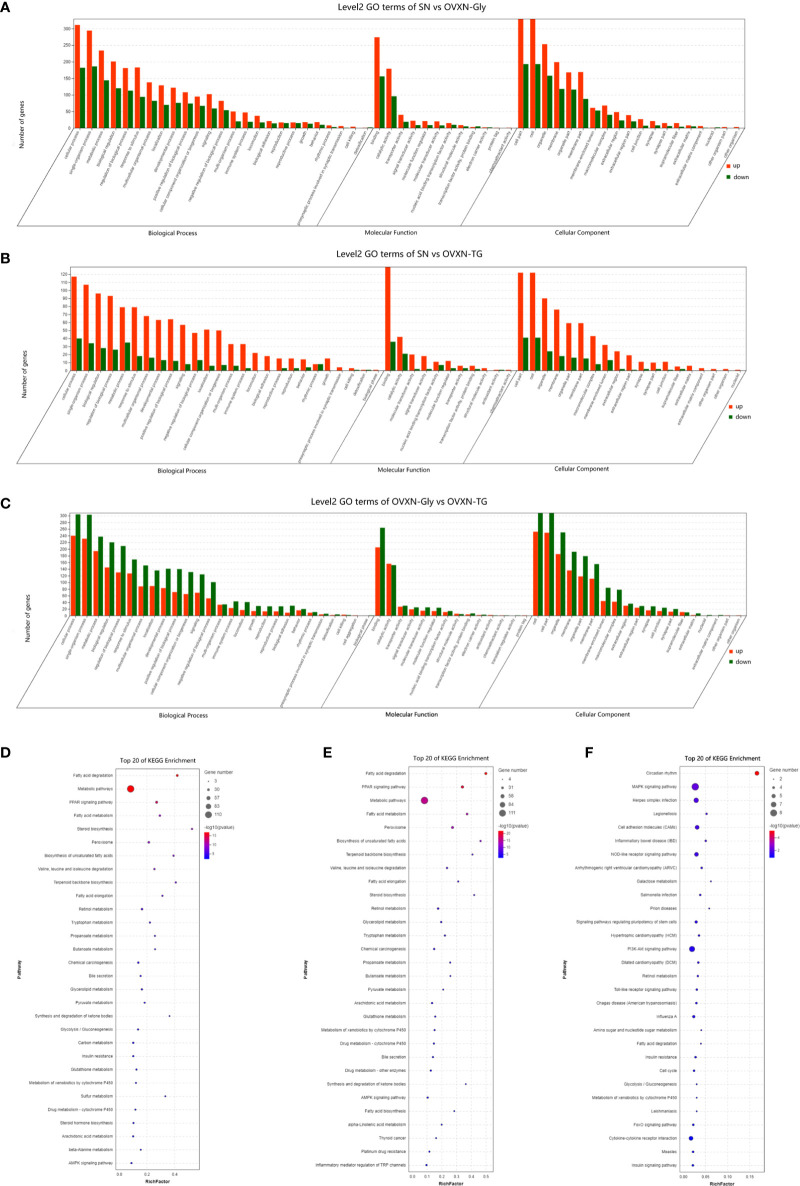
Enriched molecular functions and biological pathways of the differentially expressed genes in the liver of the ovariectomized and control mice fed with NFD. **(A–C)** GO classification of the source genes of the differentially expressed mRANs in the SN *vs*. OVXN-Gly, SN *vs*. OVXN-TG and OVXN-Gly *vs*. OVXN-TG groups respectively. **(D–F)** KEGG assignments of the source genes of the differentially expressed mRANs in the SN *vs*. OVXN-Gly, OVXN-Gly *vs*. OVXN-TG and SN *vs*. OVXN-TG groups respectively. SN, sham operated control mice fed with normal food diet; OVXN-Gly, ovariectomized mice fed with normal food diet and glycogen accumulated in its liver; OVXN-TG, ovariectomized mice fed with normal food diet and triglyceride accumulated in its liver.

**Figure 5:**
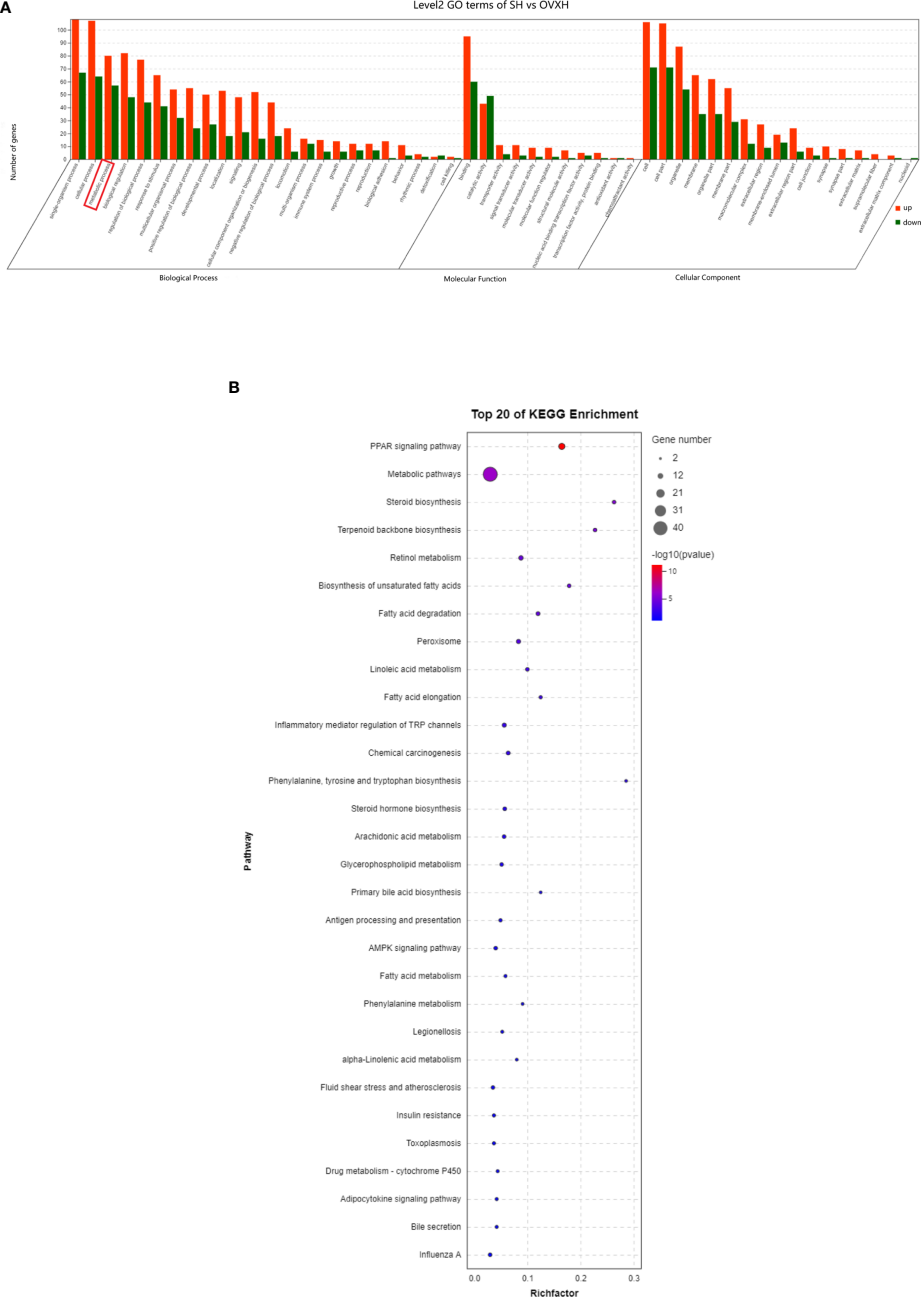
GO classification and KEGG assignments to enrich molecular functions and biological pathways of the differentially expressed genes in the liver of the ovariectomized and control mice fed with HFD. **(A)** GO classification of the source genes of the differentially expressed mRANs in the SH and OVXH groups. **(B)** KEGG assignments of the source genes of the differentially expressed mRANs in the SH and OVXH groups. SH, sham operated control mice fed with high fat diet; OVXH, ovariectomized mice fed with high fat diet.

The GO classification results showed that metabolic process was significantly affected by the ovariectomy in mice fed with NFD or HFD (**Figures 4A–C**, **5A**, **S5B**, **S6C, and D**). The results of KEGG assignments demonstrated that there were 11 pathways, including “Fatty acid degradation”, “Metabolic pathways”, “PPAR signaling pathway”, “Fatty acid metabolism”, “Steroid biosynthesis”, “Peroxisome”, “Biosynthesis of unsaturated fatty acids”, “Terpenoid backbone biosynthesis”, “Fatty acid elongation”, “Retinol metabolism “and “Chemical carcinogenesis”, significantly altered between the SN *vs*. OVXN-Gly groups (**Figure 4D**) or the SH *vs*. OVXH groups (**Figure 5B**). The pathways of “Circadian rhythm”, “Insulin resistance”, “Glycolysis/Gluconeogenesis” and “Insulin signal pathway” were also altered in mice of the OVXN-TG group compared with the SN group (**Figure 4F**). These results illustrated that hepatic metabolic pathways, especially fatty acid metabolism and PPAR signaling pathway, were significantly affected by ovariectomy in mice. The results of KEGG assignments for OVXN-Gly *vs*. OVXN-TG groups, SN *vs*. SH groups, OVXN-Gly *vs*. OVXH groups and OVXN-TG *vs*. OVXH groups were shown in **Figures 4E**, **S5C**, **S6E, and F**, respectively.

### Ovariectomy Altered the Composition of Gut Microbiota in Mice

Since the gut microbiota had very important roles in regulating the hepatic metabolism (48, 49), in the present study, the 16S rDNA genes of the gut microbiota from the ovariectomized and control mice were sequenced (https://www.ncbi.nlm.nih.gov/sra/PRJNA699121). The Shannon rarefaction curves of 4 groups all reached the saturation plateau (**Figure S7A**), demonstrating enough sequence coverage of fecal samples to describe the composition of gut microbiota. The principal coordinates analysis (PCoA) illustrated that the samples from the 4 groups could be distinguished clearly (**Figure S7B**). The Venn diagram showed that there were 256 common OTUs in the four groups of mice, and there were 183, 113, 661 and 205 OTUs specific to the mice of the SN, OVXN, SH and OVXH groups, respectively (**Figure S7C**).

**Figure 6:**
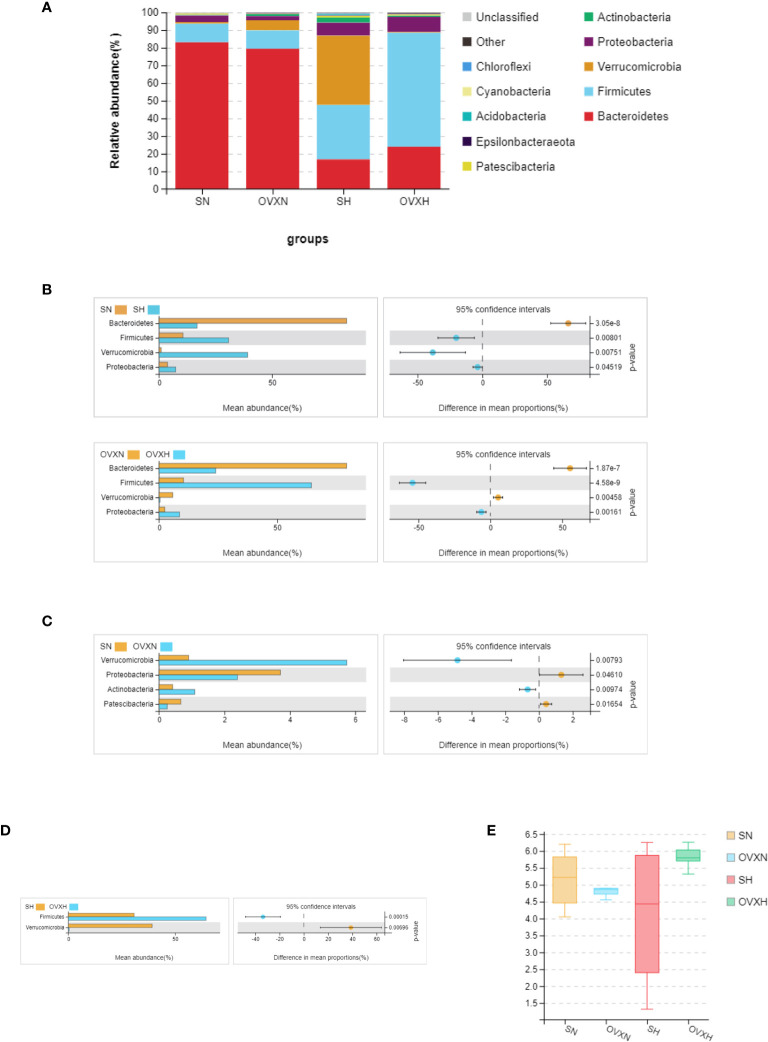
Relative abundance of the gut microbiota of the ovariectomized and control mice with NFD or HFD. **(A)** Relative abundance of gut microbiota at phylum levels of the SN, OVXN, SH and OVXH groups. Different colors were used to demonstrate each flora. **(B)** Different abundance of gut microbiota at phylum levels of SN *vs*. SH and OVXN *vs*. OVXH groups respectively. **(C)** Different abundance of gut microbiota at phylum levels between the SN and OVXN groups. **(D)** Different abundance of gut microbiota at phylum levels between the SH and OVXH groups. **(E)** The Shannon index of α diversity of the gut microbiota from the SN, OVXN, SH and OVXH groups. SN, sham operated control mice fed with normal food diet; SH, sham operated control mice fed with high fat diet; OVXH, ovariectomized mice fed with high fat diet; OVXN, ovariectomized mice fed with normal food diet.

All the sequences were classified from phylum to species levels, and the taxonomic compositions of the bacterial phylum of the SN, OVXN, SH and OVXH groups were shown in **Figure 6A**. On the phylum level, compared with the SN group, the SH group had higher *Firmicutes*, *Verrucomicrobia*, and *Proteobacteria*, but lower *Bacteroidetes* (**Figures 6A, B**). For OVX mice the abundances of *Firmicutes* and *Proteobacteria* were significantly higher, whereas the abundance of *Bacteroidetes* and *Verrucomicrobia* was significantly lower in the OVXH group compared with the OVXN group (**Figures 6A, B**). These results were similar to other reports that HFD could increase *Firmicutes* and *Proteobacteria* and decrease *Bacteroidetes* (50, 51). The *Verrucomicrobia* and *Actinobacteria* were significantly increased in the OVXN group compared with the SN group (**Figure 6C**), and the *Proteobacteria* and *Patescibacteria* were significantly decreased in the OVXN group compared with the SN group (**Figure 6C**). In mice fed with HFD, the relative abundance of *Firmicutes* was significantly increased in the OVXH group, whereas the relative abundance of *Verrucomicrobia* was significantly decreased compared with the SH group (**Figure 6D**). The Shannon index of α diversity showed that the bacterial diversity of the OVXN group was reduced compared with the SN group, however, the bacterial diversity of the OVXH group was the highest in the four groups (**Figure 6E**). These results demonstrated that both ovariectomy and HFD could affect the composition of gut microbiota and *Firmicutes* was induced to increase more easily by HFD in ovariectomized mice than in sham mice. The taxonomic compositions of the four groups on class and family levels were presented in **Figures S8A, B**.

According to the results of the LEFse analysis, 20 bacterial taxa differed in abundance between the SN and SH groups, with 11 bacterial taxa distinctive for the SN group, and 9 bacterial taxa distinctive for the SH group (**Figures S8C, D**). There were 43 bacterial taxa differed in abundance between the OVXN and the OVXH groups, with 15 bacterial taxa distinctive for the OVXN group, including *Burkholderales_bacterium_YL45* and *Parasutterella*, 28 bacterial taxa distinctive for the OVXH group, including *Lachnospiraceae*, *Lachnospiraceae_bacterium_615* and *Lachnospiraceae_bacterium_28_4* (**Figures S8E, F**). There were 3 bacterial taxa differed in abundance between the SN and OVXN groups (**Figures S9A, B**); *Lactobacillus_murinus* and *Rs_E47_termite_group* were biomarkers for the SN group, and *Burkholderales_bacterium_YL45* was predominant in the OVXN group (**Figures S9A, B**). *Lachnospiraceae* and *Parasutterella* were biomarkers for the OVXH group compared with the SH group (**Figures S9C, D**).

**Figure 7:**
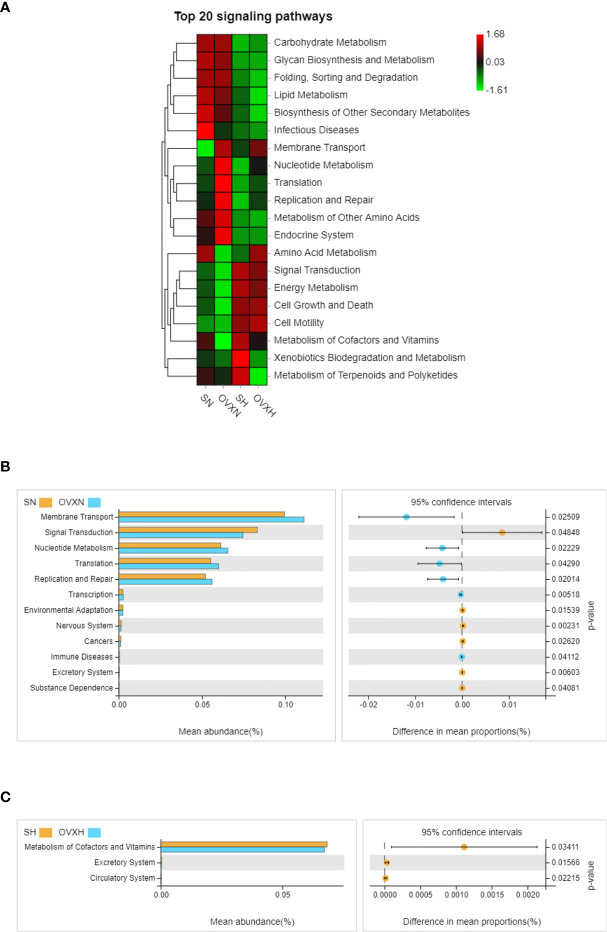
KEGG enrichment analysis of the altered signaling pathways based on the relative abundance of the gut microbiota of the ovariectomized and control mice with NFD or HFD. **(A)** Heatmap of top 20 signaling pathways in the SN, OVXN, SH and OVXH groups. **(B)** Presentation of the altered KEGG pathways in the SN and OVXN groups. **(C)** Presentation of the altered KEGG pathways in the SH and OVXH groups (bar plots on the left side displayed the meant proportion of each KEGG pathway. Dot plots on the right side showed the differences in mean proportions between the two indicated groups using q-values). KEGG, Kyoto Encyclopedia of Genes and Genomes; SN, sham operated control mice fed with normal food diet; OVXN, ovariectomized mice fed with normal food diet; SH, sham operated control mice fed with high fat diet; OVXH, ovariectomized mice fed with high fat diet.

The KEGG pathway analysis was used to predict the functional profiles of the altered gut microbiota. Based on the alteration of the composition of gut microbiota, 12 pathways were predicted to be significantly affected between the SN group and the OVXN group (**Figures 7A, B**), including “Membrane Transport”, “Signal Transduction”, “Nucleotide Metabolism”, “Translation”, “Replication and Repair”, “Transcription”, “Environmental Adaptation”, “Nervous System”, “Cancers”, “Immune Diseases”, “Excretory System” and “Substance Dependence”. For the SH group and OVXH group, 3 pathways were predicted to be significantly affected, including “Metabolism of Cofactors and Vitamins”, “Excretory System” and “Circulatory System” (**Figures 7A, C**). The results of KEGG signaling pathway analysis for the SN *vs* SH groups and OVXN *vs* OVXH groups were shown in **Figures S10A, B**, respectively.

## Discussion

Herein, ovariectomized mice fed with NFD or HFD were used to study the functions of ovaries in regulating hepatic glucose and lipid metabolism and gut microbiota. The body weight and the serum content of LDL-C were both significantly increased after ovariectomy. The storage of glycogen or TG was also increased in the liver of ovariectomized mice. The results of transcriptome analysis showed that the expression level of many genes related with glucose and lipid metabolism was changed in the liver of ovariectomized mice. The expression of genes related with fatty acid and triacylglycerol synthesis, genes related with cholesterol metabolism, and PPARα and its targeted genes were confirmed through qPCR and WB assays. The composition of the gut microbiota in mice was also changed by ovariectomy and HFD. In summary, ovariectomy broke the homeostasis of hepatic metabolism and altered the composition of gut microbiota in mice fed with NFD or HFD.

LDL-C was one of the major risk factors of the cardiovascular disease (CVD) (52). So, the increase of plasma TC in the OVXN mice and the increase of serum LDL-C in both the OVXN and the OVXH mice (**Figure 1D**) indicated the increase of risk factors of CVD in the ovariectomized mice. Hence, the ovariectomized mice used in the present study would be useful for the discovery of new drugs to reduce the serum TC and LDL-C concentration in midlife and elderly females.

There was no significant change of the serum concentration of fasting glucose, TG, HDL-C, ALT and AST in ovariectomized mice compared with the sham operation control mice in the present study, the reason may be because the effect of ovariectomy and HFD on these biochemical indexes was still slight within 6 weeks. It has been reported that the plasma TC and LDL-C were increased in the aging humans and rodents since their physiological function in eliminating cholesterol and LDL-C decreased (53). We speculated that the increase of TC in the OVXN mice and the increase of LDL-C in both the OVXN and the OVXH mice was also caused by the decrease of the physiological function in eliminating them. The situation of normal fasting glucose and normal TG concentration in serum of hypercholesterolemia patients have also been reported previously (54–57). It indicated that ovaries might have very important functions in eliminating TC and LDL-C in females. So, low cholesterol containing diet was recommended for the menopausal women, and the moderate dose of drugs which reduce serum LDL-C should be used for cases with serious symptoms.

In the present study, glycogen was found to be accumulated in the liver of around one third of ovariectomized mice fed with NFD, and the other mice in this group showed lipid accumulation in the liver. However, large lipid drops appeared in the liver of all the ovariectomized mice fed with HFD. Abnormal accumulation of glycogen and lipid could lead to liver damage (58), and long-term accumulation of them even might cause hepatocellular carcinoma (HCC) or hepatocellular adenoma (HCA) in patients with glycogen storage disease (GSD) (59, 60). Allende DS et al. (61) reported that excess glycogen accumulation in liver caused greater hepatocellular injury than that of lipid accumulation in humans, and it might be the important reason why the liver weight and liver index in the OVXN-Gly mice were higher than those in the OVXN-TG mice in the present study (**Figure 2C**). Ahmed-Sorour et al. (62) reported that the glycogen deposition in liver, uterus, skeletal and cardiac muscle was reduced in female mice after 16 weeks of ovariectomy. In the present study, the excessive glycogen deposition was found in the liver of female mice after 6 weeks of ovariectomy. It might be the difference between the short-term and long-term effect of ovariectomy. Recent study demonstrated that ovariectomy promoted glucocorticoid (GC) hypersensitivity in the liver of mice, and hepatic glucocorticoid receptor (GR) pathway was a driver of steatosis in ovariectomized females (33). Kuo et al. (63) reported that GC could increase glycogen storage in liver *via* inducing the activity of glycogen synthase. We speculated that GC promoted the glycogen deposition in the liver of mice within the short-term of ovariectomy, and the excessive glycogen was converted to lipid in the liver of ovariectomized mice, because the expression levels of genes related with lipogenesis, such as Fasn, Acca1 and Elovl6, were significantly upregulated in the OVXN-Gly group compared with other groups in the present study (**Figure 3B**). Quinn et al. (33) found the fatty livers of OVX mice after 3 months of the operation. However, in the current research, the liver tissues were collected only after 6 weeks of the operation. At the time point of 6 weeks, the conversion from accumulated glycogen to lipids in hepatocytes was still not completed in a small part of mice from the OVXN group, although most of mice in OVXN group showed lipids accumulated in hepatocytes at this stage. Hence, two different histological results were found in the OVXN group of the present study. HFD might accelerate the conversion of glycogen to lipids, so there were only excessive lipid drops were detected in the liver of the OVXH mice (**Figure 2A**).

The expression of Cyp3a41a mRNA was almost lost in the liver of ovariectomized mice (**Figure 3A**). Cyp3a41a was one of the female-specific members of the CYP3A gene subfamily in the mouse liver, and estradiol could induce its expression in the liver of adult female mice (46). The loss of Cyp3a41a mRNA might be caused by the reduction of estradiol and other hormones mainly produced by ovaries in the circulation of ovariectomized mice. The results of transcriptome and qPCR indicated that ovariectomy caused the alteration of the expression of many genes related with glucose and lipid metabolism in the liver. Since the expression of estrogen receptor (ER) β was undetectable in the mouse liver (33), ERα was the most important pathway through which estrogen regulated the expression of genes in liver. Gao et al. (35) found that many ERα binding sites were enriched in the promoters of genes involved in glucose and lipid metabolism, including Ptgds, Acox1, Acox2, Cpt1a, Pck1, Mapk14, Pparα and Pgc-1. PPARα was one of the key transcriptional factors in hepatic β-oxidation (64). In the present study, although the mRNA level of PPARα was not significantly reduced, the expression levels of its target genes including Cyp4a10, Cyp4a14 and Cyp4a31 were significantly downregulated in the OVXN-Gly group (**Figures 3A, C**), it might be caused by the reduction of the activity of PPARα. The reduction of the activity of PPARα might be one of the reasons of the disorders of glucose and lipid metabolism in the liver of ovariectomized mice.

Results of 16S rDNA analysis indicated that the composition of gut microbiota was altered after ovariectomy of mice fed with NFD or HFD. Ovariectomy significantly increased the *Firmicutes* in the gut of ovariectomized mice fed with HFD compared with the sham control mice fed with HFD. Since high relative abundance of *Firmicutes* was related to glucose and lipid metabolic disorders in human and rodents (65, 66), it might indicate that ovariectomy exacerbated obesity caused by HFD. Recent study demonstrated that depletion of Treg cells could increase the relative abundance of *Firmicutes* in the gut of mice (67). So, we guess that ovariectomy would affect the immune homeostasis of the intestinal mucosa, especially for individuals with HFD. The results of LEFse analysis confirmed that *Lachnospiraceae* was predominant in the OVXH group compared with the SH and OVXN groups. Kameyama et al. (68) reported that one *Lachnospiraceae* bacterium (strain AJ110941) could induce obesity and diabetes in ob/ob mice. Different taxa of *Lachnospiraceae* were related with different extra- and intra-intestinal diseases (69). It also indicated that ovariectomy exacerbated metabolic disorders and other diseases which were associated with HFD. So, prebiotics or probiotics were suggested for menopausal women, especially for midlife and elder women with HFD, to improve the hepatic glucose and lipid metabolic disorders.

**Figure 8:**
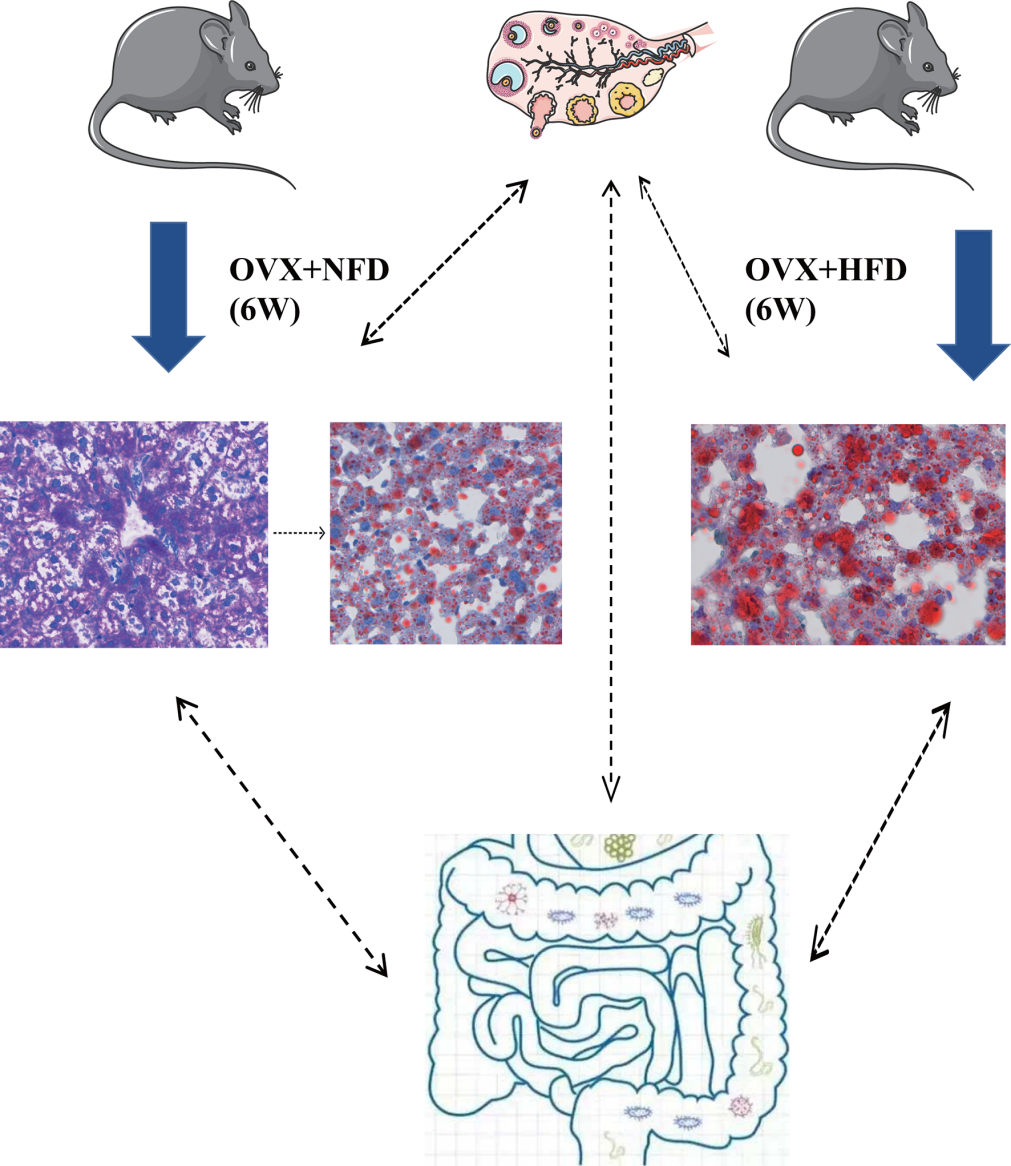
Ovariectomy impaired hepatic glucose and lipid homeostasis and altered the gut microbiota in mice. Ovariectomy caused the increase of serum LDL-C and excessive storage of glycogen and lipids in hepatocytes and also altered the gut microbiota in female mice fed with NFD or HFD. Ovariectomy could promote the glycogen deposition in hepatocytes within the short term after operation, and then the excessive glycogen was converted to lipid; high fat diet might accelerate the conversion of glycogen to lipids in the hepatocytes of ovariectomized mice. NFD, normal food diet; HFD, high fat diet.

In conclusion, ovariectomy caused the increase of serum LDL-C, excessive storage of glycogen and lipids in hepatocytes and also altered the gut microbiota in female mice (**Figure 8**). The present study demonstrated the important functions of hormones generated by female ovaries on keeping the homeostasis of glucose and lipid metabolism. These hormones might regulate the hepatic glucose and lipid metabolism through multiple pathways directly or indirectly. The excessive accumulated glycogen in the liver of ovariectomized mice was first found in the current research, and it might be one of the mechanisms of female liver injuries caused by ovaries aging. Analyzing the hepatic metabolism of ovariectomized mice at different time points after operation and the transplantation of gut microbiota between ovariectomized and sham operated mice will be conducted in our future research to uncover the detailed mechanism of ovary generated hormones on regulating the homeostasis of glucose and lipid metabolism in females. Ovariectomized mice would be helpful to develop drugs for prevention and treatment of hepatic glucose and lipid metabolic disorders of menopausal women.

## Data Availability Statement

The data presented in the study are deposited in the SRA repository, accession number PRJNA699121. (NCBI SRA database, accession number: PRJNA699121) can be found in the section of results in the article.

## Ethics Statement

The animal study was reviewed and approved by the animal ethics committee of Guangdong Pharmaceutical University. Written informed consent was obtained from the owners for the participation of their animals in this study.

## Author Contributions

ZL, YY and JG: Designed the study and conceived the report. HW and YY: Analyzed and interpreted the results of mRNA sequencing. YY and ZL: Wrote the draft of the manuscript and revised it critically. HW, QH, WL and YN: Established the mouse model. HW and YL: Analyzed and interpreted the results of fecal 16rDNA sequencing. HW, YY, QH, YL, WL, YN, LY, XZ, CY, TL, FT and JZ: Performed the biochemical and molecular experiments, including serum biochemical profile, Western blot, qPCR, H&E staining, Oil Red O staining and PAS staining. HW, YL and FT: Created the figures and tables. All authors had read and approved the final manuscript.

## Funding

This work was supported by the National Natural Science Foundation of China (No. 81830113, No. 81803912, No. 81530102, No. 31671520), National key R & D plan “Research on modernization of traditional Chinese medicine” (2018YFC1704200), Major basic and applied basic research projects of Guangdong Province of China (2019B030302005), the Opening Foundation of the Key Laboratory of Regenerative Biology, Guangzhou Institutes of Biomedicine and Health, Chinese Academy of Sciences (KLRB201807), the Science and Technology Planning Project of Guangzhou City (No. 201803010069), the Characteristic Innovation Project (Natural Science) of the Education Department of Guangdong Province and the “Innovation Strong School Project” of Guangdong Pharmaceutical University (No. 2017KTSCX102), and the Science and Technology Project of Yue-Xiu District of Guangzhou (No. 2018-WS-011).

## Conflict of Interest

The authors declare that the research was conducted in the absence of any commercial or financial relationships that could be construed as a potential conflict of interest.

## Supplementary Material

Supplementary Figure 1 The average food intake and the serum concentrations of glucose, TG, ALT and AST of mice with NFD or HFD. **(A)** The average daily food intake within 6weeks after operation of mice from the SN, OVXN, SH and OVXH groups. **(B–E)** The concentration of fasting plasm glucose (FPG), TG, ALT, and AST in the serum of mice from the SN, OVXN, SH and OVXH groups at six weeks after operation. SN, sham operated control mice fed with normal food diet; OVXN, ovariectomized mice fed with normal food diet; SH, sham operated control mice fed with high fat diet; OVXH, ovariectomized mice fed with high fat diet.

Supplementary Figure 2 Transcriptome analysis of the liver from ovariectomized and control mice with NFD or HFD. **(A)** Statistics of differentially expressed genes in the liver of mice compared between every group of SN, OVXN-Gly, OVXN-TG, SH and OVXH. **(B)** Heatmap and volcano pot showed differentially expressed genes in the liver of mice from the SN and OVXN-Gly groups. **(C)** Heatmap and volcano pot showed differentially expressed genes in the liver of mice from the SH and OVXH groups. **(D)** Heatmap and volcano pot showed differentially expressed genes in the liver of mice from the OVXN-Gly and OVXN-TG groups. **(E)** Heatmap and volcano pot showed differentially expressed genes in the liver of mice from the SN and OVXN-TG groups. SN, sham operated control mice fed with normal food diet; OVXN-Gly, ovariectomized mice fed with normal food diet and glycogen accumulated in its liver; OVXN-TG, ovariectomized mice fed with normal food diet and triglyceride accumulated in its liver; SH, sham operated control mice fed with high fat diet; OVXH, ovariectomized mice fed with high fat diet.

Supplementary Figure 3 Relationship analysis of liver tissues of the ovariectomized and control mice with NFD or HFD for transcriptional sequences. **(A)** Heatmap demonstrated the relationship of liver tissues of the SN, OVXN, SH and OVXH groups. **(B)** PCA analysis of the relationship of liver tissues of the SN, OVXN, SH and OVXH groups. SN, sham operated control mice fed with normal food diet; OVXN, ovariectomized mice fed with normal food diet; SH, sham operated control mice fed with high fat diet; OVXH, ovariectomized mice fed with high fat diet.

Supplementary Figure 4 Transcriptome analysis showing differentially expressed genes of the liver tissues from the SN and OVXN-TG mice. **(A)** Statistics of differentially expressed genes in the liver of the SN and OVXN-TG groups. **(B)** Volcano pot showed differentially expressed genes in liver of the SN and OVXN-TG groups. SN, sham operated control mice fed with normal food diet; OVXN-TG, ovariectomized mice fed with normal food diet and triglyceride accumulated in its liver.

Supplementary Figure 5 Enriched molecular functions and biological pathways of the differentially expressed genes in the liver tissues of the SN and SH groups. **(A)** Heatmap and volcano pot showed differentially expressed genes in liver of the SN and SH groups. **(B)** GO classification of the source genes of the differentially expressed mRANs in the liver of the SN and SH groups. **(C)** KEGG assignments of the differentially expressed mRNAs in the liver of the SN and SH groups. SN, sham operated control mice fed with normal food diet; SH, sham operated control mice fed with high fat diet.

Supplementary Figure 6 Enriched molecular functions and biological pathways of the differentially expressed genes in the liver tissues of the OVXN and OVXH groups. **(A)** Heatmap and volcano pot showed differentially expressed genes in liver of the OVXN-Gly and OVXH groups. **(B)** Heatmap and volcano pot showed differentially expressed genes in liver of the OVXN-TG and OVXH groups. **(C, D)** GO classification of the source genes of the differentially expressed mRNAs in the liver of the OVXN-Gly *vs*. OVXH and OVXN-TG *vs*. OVXH respectively. **(E, F)** KEGG assignments of the differentially expressed mRNAs in the liver of the OVXN-Gly *vs*. OVXH and OVXN-TG *vs*. OVXH groups, respectively. OVXN-Gly, ovariectomized mice fed with normal food diet and glycogen accumulated in its liver; OVXN-TG, ovariectomized mice fed with normal food diet and triglyceride accumulated in its liver; OVXH, ovariectomized mice fed with high fat diet.

Supplementary Figure 7 Overview of 16S rDNA genes of the gut microbiota of the ovariectomized and control mice with NFD or HFD. **(A)** Shannon rarefaction curves for the SN, OVXN, SH, and OVXH groups. **(B)** PCoA analysis of the gut microbiota of the SN, OVXN, SH and OVXH groups. **(C)** Venn diagram showed the number of the OTUs of the SN, OVXN, SH and OVXH groups. SN, sham operated control mice fed with normal food diet; OVXN, ovariectomized mice fed with normal food diet; SH, sham operated control mice fed with high fat diet; OVXH, ovariectomized mice fed with high fat diet; OTU, operational taxonomic units; PCoA, principal co-ordinates analysis.

Supplementary Figure 8 Relative abundance of the gut microbiota of the SN, OVXN, SH and OVXH groups. **(A, B)** Relative abundance of gut microbiota at class and family levels of the SN, OVXN, SH and OVXH groups. Different colors were used to demonstrate each flora. **(C, D)** LDA scores and cladogram illustrated the predominant abundant taxons of the SN and SH groups. Red represented the bacteria distinctive for the SH group and green represented the bacteria distinctive for the SN group. **(E, F)** LDA scores and cladogram illustrated the predominant abundant taxons of the OVXN and OVXH groups. Red represented the bacteria distinctive for the OVXH group and green represented the bacteria distinctive for the OVXN group. SN, sham operated control mice fed with normal food diet; OVXN, ovariectomized mice fed with normal food diet; SH, sham operated control mice fed with high fat diet; OVXH, ovariectomized mice fed with high fat diet; LDA, Linear Discriminant Analysis.

Supplementary Figure 9 LEFse analysis indicated bacterial taxa difference in abundance of gut microbiota of the ovariectomized and control mice with NFD or HFD. **(A, B)** LDA scores and cladogram illustrated the predominant abundant taxons of the SN and OVXN groups. Red represented the bacteria distinctive for the OVXN group and green represented the bacteria distinctive for the SN group. **(C, D)** LDA scores and cladogram illustrated the predominant abundant taxons of the SH and OVXH groups. Red represented the bacteria distinctive for the OVXH group and green represented the bacteria distinctive for the SH group. SN, sham operated control mice fed with normal food diet; OVXN, ovariectomized mice fed with normal food diet; SH, sham operated control mice fed with high fat diet; OVXH, ovariectomized mice fed with high fat diet; LDA, Linear Discriminant Analysis.

Supplementary Figure 10 KEGG enrichment analysis of the altered signaling pathways based on the relative abundance of the gut microbiota of the SN, OVXN, SH, OVXH groups. **(A)** Presentation of the altered KEGG pathways in the SN and SH groups. **(B)** Presentation of the altered KEGG pathways in the OVXN and OVXH groups (bar plots on the left side displayed the mean proportion of each KEGG pathway. Dot plots on the right side showed the differences in mean proportions between the two indicated groups using q-values). KEGG, Kyoto Encyclopedia of Genes and Genomes; SN, sham operated control mice fed with normal food diet; OVXN, ovariectomized mice fed with normal food diet; SH, sham operated control mice fed with high fat diet; OVXH, ovariectomized mice fed with high fat diet.

## References

[1] Chao HW Chao SW Lin H Ku HC Cheng CF. Homeostasis of Glucose and Lipid in Non-Alcoholic Fatty Liver Disease. Int J Mol Sci (2019) 20:298. 10.3390/ijms20020298 PMC6359196 30642126

[2] Atawia RT Bunch KL Toque HA Caldwell RB Caldwell RW. Mechanisms of Obesity-Induced Metabolic and Vascular Dysfunctions. Front Biosci (Landmark edition) (2019) 24:890–934. 10.2741/4758 PMC6689231 30844720

[3] Palmisano BT Zhu L Eckel RH Stafford JM. Sex Differences in Lipid and Lipoprotein Metabolism. Mol Metab (2018) 15:45–55. 10.1016/j.molmet.2018.05.008 29858147 PMC6066747

[4] Gerdts E Regitz-Zagrosek V. Sex Differences in Cardiometabolic Disorders. Nat Med (2019) 25:1657–66. 10.1038/s41591-019-0643-8 31700185

[5] Tramunt B Smati S Grandgeorge N Lenfant F Arnal JF Montagner A. Sex Differences in Metabolic Regulation and Diabetes Susceptibility. Diabetologia (2020) 63:453–61. 10.1007/s00125-019-05040-3 PMC6997275 31754750

[6] Chella Krishnan K Mehrabian M Lusis AJ. Sex Differences in Metabolism and Cardiometabolic Disorders. Curr Opin lipidol (2018) 29:404–10. 10.1097/MOL.0000000000000536 PMC6382080 30156571

[7] Mauvais-Jarvis F Manson JE Stevenson JC Fonseca VA. Menopausal Hormone Therapy and Type 2 Diabetes Prevention: Evidence, Mechanisms, and Clinical Implications. Endocrine Rev (2017) 38:173–88. 10.1210/er.2016-1146 PMC5460681 28323934

[8] Tiano JP Mauvais-Jarvis F. Importance of Oestrogen Receptors to Preserve Functional β-Cell Mass in Diabetes. Nat Rev Endocrinol (2012) 8:342–51. 10.1038/nrendo.2011.242 22330739

[9] Carr MC. The Emergence of the Metabolic Syndrome With Menopause. J Clin Endocrinol Metab (2003) 88:2404–11. 10.1210/jc.2003-030242 12788835

[10] Mauvais-Jarvis F. Estrogen and Androgen Receptors: Regulators of Fuel Homeostasis and Emerging Targets for Diabetes and Obesity. Trends Endocrinol Metabol: TEM (2011) 22:24–33. 10.1016/j.tem.2010.10.002 PMC3011051 21109497

[11] Mauvais-Jarvis F Clegg DJ Hevener AL. The Role of Estrogens in Control of Energy Balance and Glucose Homeostasis. Endocrine Rev (2013) 34:309–38. 10.1210/er.2012-1055 PMC3660717 23460719

[12] Park SK Harlow SD Zheng H Karvonen-Gutierrez C Thurston RC Ruppert K. Association Between Changes in Oestradiol and Follicle-Stimulating Hormone Levels During the Menopausal Transition and Risk of Diabetes. Diabetic Med: J Br Diabetic Assoc (2017) 34:531–8. 10.1111/dme.13301 PMC5352524 27973745

[13] Quinn MM Cedars MI. Cardiovascular Health and Ovarian Aging. Fertil Sterility (2018) 110:790–3. 10.1016/j.fertnstert.2018.07.1152 30316413

[14] Towfighi A Zheng L Ovbiagele B. Sex-Specific Trends in Midlife Coronary Heart Disease Risk and Prevalence. Arch Internal Med (2009) 169:1762–6. 10.1001/archinternmed.2009.318 19858433

[15] Stefanska A Bergmann K Sypniewska G. Metabolic Syndrome and Menopause: Pathophysiology, Clinical and Diagnostic Significance. Adv Clin Chem (2015) 72:1–75. 10.1016/bs.acc.2015.07.001 26471080

[16] Hong YC. Aging Society and Environmental Health Challenges. Environ Health Perspect (2013) 121:A68–9. 10.1289/ehp.1206334 PMC3621200 23454410

[17] Liu H Zhou C Qi D Gao Y Zhu M Tao T. Inhibiting Monoacylglycerol Lipase Suppresses RANKL-Induced Osteoclastogenesis and Alleviates Ovariectomy-Induced Bone Loss. Front Cell Dev Biol (2021) 9:640867. 10.3389/fcell.2021.640867 33777947 PMC7994615

[18] Cho KM Kim YS Lee M Lee HY Bae YS. Isovaleric Acid Ameliorates Ovariectomy-Induced Osteoporosis by Inhibiting Osteoclast Differentiation. J Cell Mol Med (2021) 25:4287–97. 10.1111/jcmm.16482 PMC8093970 33768674

[19] Wang X Wang M Cui X Li Z Guo S Gao F. Antiosteoporosis Effect of Geraniin on Ovariectomy-Induced Osteoporosis in Experimental Rats. J Biochem Mol Toxicol (2021) 35 1–8. 10.1002/jbt.22774 33755276

[20] Rodrigues FM Adélio JI Santana VO De Marco Ornelas E de Souza RR Cardoso CG. Physical Exercise Alters Hepatic Morphology of Low-Density Lipoprotein Receptor Knockout Ovariectomized Mice. Med Mol Morphol (2019) 52:15–22. 10.1007/s00795-018-0198-7 29934711

[21] Kim J Lee H Lim J Lee H Yoon S Shin SS. The Lemon Balm Extract ALS-L1023 Inhibits Obesity and Nonalcoholic Fatty Liver Disease in Female Ovariectomized Mice. Food Chem Toxicol (2017) 106:292–305. 10.1016/j.fct.2017.05.059 28571771

[22] Tuazon MA Campbell SC Klein DJ Shapses SA Anacker KR Anthony TG. Effects of Ovariectomy and Exercise Training Intensity on Energy Substrate and Hepatic Lipid Metabolism, and Spontaneous Physical Activity in Mice. Metabol: Clin Exp (2018) 83:234–44. 10.1016/j.metabol.2018.02.011 29522773

[23] Han HS Kang G Kim JS Choi BH Koo SH. Regulation of Glucose Metabolism From a Liver-Centric Perspective. Exp Mol Med (2016) 48:e218. 10.1038/emm.2015.122 26964834 PMC4892876

[24] Gonçalves DC Lira FS Yamashita AS Carnevali Junior LC Eder R Laviano A. Liver Lipid Metabolism Disruption in Cancer Cachexia Is Aggravated by Cla Supplementation -Induced Inflammation. Clin Nutr (Edinburgh Scotland) (2019) 38:2219–30. 10.1016/j.clnu.2018.09.023 30322784

[25] Ding HR Wang JL Ren HZ Shi XL. Lipometabolism and Glycometabolism in Liver Diseases. BioMed Res Int (2018) 2018:1287127. 10.1155/2018/1287127 31205932 PMC6530156

[26] Kim JK Gavrilova O Chen Y Reitman ML Shulman GI. Mechanism of Insulin Resistance in a-ZIP/F-1 Fatless Mice. J Biol Chem (2000) 275:8456–60. 10.1074/jbc.275.12.8456 10722680

[27] Petersen KF Dufour S Befroy D Lehrke M Hendler RE Shulman GI. Reversal of Nonalcoholic Hepatic Steatosis, Hepatic Insulin Resistance, and Hyperglycemia by Moderate Weight Reduction in Patients With Type 2 Diabetes. Diabetes (2005) 54:603–8. 10.2337/diabetes.54.3.603 PMC2995496 15734833

[28] Fabbrini E Magkos F Mohammed BS Pietka T Abumrad NA Patterson BW. Intrahepatic Fat, Not Visceral Fat, Is Linked With Metabolic Complications of Obesity. Proc Natl Acad Sci USA (2009) 106:15430–5. 10.1073/pnas.0904944106 PMC2741268 19706383

[29] Samuel VT Shulman GI. Mechanisms for Insulin Resistance: Common Threads and Missing Links. Cell (2012) 148:852–71. 10.1016/j.cell.2012.02.017 PMC3294420 22385956

[30] Jackson KC Wohlers LM Valencia AP Cilenti M Borengasser SJ Thyfault JP. Wheel Running Prevents the Accumulation of Monounsaturated Fatty Acids in the Liver of Ovariectomized Mice by Attenuating Changes in SCD-1 Content. Appl Physiol Nutr Metab = Physiologie Appliquee Nutr Metabolisme (2011) 36:798–810. 10.1139/h11-099 22026420

[31] Oliveira MC Campos-Shimada LB Marçal-Natali MR Ishii-Iwamoto EL Salgueiro-Pagadigorria CL. A Long-Term Estrogen Deficiency in Ovariectomized Mice Is Associated With Disturbances in Fatty Acid Oxidation and Oxidative Stress. Rev Bras ginecologia e obstetricia: Rev da Federacao Bras das Sociedades Ginecologia e Obstetricia (2018) 40:251–9. 10.1055/s-0038-1666856 PMC10316894 29913542

[32] Venetsanaki V Polyzos SA. Menopause and non-Alcoholic Fatty Liver Disease: A Review Focusing on Therapeutic Perspectives. Curr Vasc Pharmacol (2019) 17:546–55. 10.2174/1570161116666180711121949 29992886

[33] Quinn MA Xu X Ronfani M Cidlowski JA. Estrogen Deficiency Promotes Hepatic Steatosis *Via* a Glucocorticoid Receptor-Dependent Mechanism in Mice. Cell Rep (2018) 22:2690–701. 10.1016/j.celrep.2018.02.041 PMC5875726 29514097

[34] Zhang Y Klein K Sugathan A Nassery N Dombkowski A Zanger UM. Transcriptional Profiling of Human Liver Identifies Sex-Biased Genes Associated With Polygenic Dyslipidemia and Coronary Artery Disease. PloS One (2011) 6:e23506. 10.1371/journal.pone.0023506 21858147 PMC3155567

[35] Gao H Fält S Sandelin A Gustafsson JA Dahlman-Wright K. Genome-Wide Identification of Estrogen Receptor Alpha-Binding Sites in Mouse Liver. Mol Endocrinol (Baltimore Md.) (2008) 22:10–22. 10.1210/me.2007-0121 PMC5419629 17901129

[36] Nicholson JK Holmes E Kinross J Burcelin R Gibson G Jia W. Host-Gut Microbiota Metabolic Interactions. Sci (New York NY) (2012) 336:1262–7. 10.1126/science.1223813 22674330

[37] Fuhrman BJ Feigelson HS Flores R Gail MH Xu X Ravel J. Associations of the Fecal Microbiome With Urinary Estrogens and Estrogen Metabolites in Postmenopausal Women. J Clin Endocrinol Metab (2014) 99:4632–40. 10.1210/jc.2014-2222 PMC4255131 25211668

[38] Cox-York KA Sheflin AM Foster MT Gentile CL Kahl A Koch LG. Ovariectomy Results in Differential Shifts in Gut Microbiota in Low Versus High Aerobic Capacity Rats. Physiol Rep (2015) 3:e12488. 10.14814/phy2.12488 26265751 PMC4562574

[39] Choi S Hwang YJ Shin MJ Yi H. Difference in the Gut Microbiome Between Ovariectomy-Induced Obesity and Diet-Induced Obesity. J Microbiol Biotechnol (2017) 27:2228–36. 10.4014/jmb.1710.10001 29121700

[40] Feng X Cheng Q Meng Q Yang Y Nie K. Effects of Ondansetron and [6]-Gingerol on Pica and Gut Microbiota in Rats Treated With Cisplatin. Drug Design Dev Ther (2019) 13:2633–41. 10.2147/DDDT.S211845 PMC6682320 31534312

[41] Yang Y Yang F Huang M Wu H Yang C Zhang X. Fatty Liver and Alteration of the Gut Microbiome Induced by Diallyl Disulfide. Int J Mol Med (2019) 44:1908–20. 10.3892/ijmm.2019.4350 PMC6777666 31573042

[42] Wang H Ren P Mang L Shen N Chen J Zhang Y. In Vitro Fermentation of Novel Microwave-Synthesized non-Digestible Oligosaccharides and Their Impact on the Composition and Metabolites of Human Gut Microbiota. J Funct Foods (2019) 55:156–66. 10.1016/j.jff.2019.02.030

[43] Shende VR Wu M Singh AB Dong B Kan CF Liu J. Reduction of Circulating PCSK9 and LDL-C Levels by Liver-Specific Knockdown of HNF1α in Normolipidemic Mice. J Lipid Res (2015) 56:801–9. 10.1194/jlr.M052969 PMC4373738 25652089

[44] Gong Y Ma Y Ye Z Fu Z Yang P Gao B. Thyroid Stimulating Hormone Exhibits the Impact on LDLR/LDL-C *Via* Up-Regulating Hepatic PCSK9 Expression. Metabol: Clin Exp (2017) 76:32–41. 10.1016/j.metabol.2017.07.006 28987238

[45] Choi E Zhang X Xing C Yu H. Mitotic Checkpoint Regulators Control Insulin Signaling and Metabolic Homeostasis. Cell (2016) 166:567–81. 10.1016/j.cell.2016.05.074 PMC5560052 27374329

[46] Sakuma T Takai M Endo Y Kuroiwa M Ohara A Jarukamjorn K. A Novel Female-Specific Member of the CYP3A Gene Subfamily in the Mouse Liver. Arch Biochem Biophys (2000) 377:153–62. 10.1006/abbi.2000.1747 10775455

[47] Zhang Y Klaassen CD. Hormonal Regulation of Cyp4a Isoforms in Mouse Liver and Kidney. Xenobiotica; Fate Foreign Compounds Biol Syst (2013) 43:1055–63. 10.3109/00498254.2013.797622 PMC4091904 23725209

[48] Brandl K Schnabl B. Intestinal Microbiota and Nonalcoholic Steatohepatitis. Curr Opin Gastroenterol (2017) 33:128–33. 10.1097/MOG.0000000000000349 PMC5662009 28257306

[49] Ma C Han M Heinrich B Fu Q Zhang Q Sandhu M. Gut Microbiome-Mediated Bile Acid Metabolism Regulates Liver Cancer *Via* NKT Cells. Sci (New York NY) (2018) 360:eaan5931. 10.1126/science.aan5931 PMC6407885 29798856

[50] Hildebrandt MA Hoffmann C Sherrill-Mix SA Keilbaugh SA Hamady M Chen YY. High-Fat Diet Determines the Composition of the Murine Gut Microbiome Independently of Obesity. Gastroenterology (2009) 137:1716–24.e1-2. 10.1097/MCO.0000000000000209 19706296 PMC2770164

[51] Murphy EA Velazquez KT Herbert KM. Influence of High-Fat Diet on Gut Microbiota: A Driving Force for Chronic Disease Risk. Curr Opin Clin Nutr Metab Care (2015) 18:515–20. 10.1097/MCO.0000000000000209 PMC4578152 26154278

[52] Mc Auley MT Mooney KM. LDL-C Levels in Older People: Cholesterol Homeostasis and the Free Radical Theory of Ageing Converge. Med Hypotheses (2017) 104:15–9. 10.1016/j.mehy.2017.05.013 28673574

[53] Han Y Do MH Kim MS Seo E Park MK Kim DK. Fenofibrate Reduces Age-Related Hypercholesterolemia in Normal Rats on a Standard Diet. Korean J Physiol Pharmacol (2010) 14:77–81. 10.4196/kjpp.2010.14.2.77 20473378 PMC2869456

[54] Berthold HK Schulte DM Lapointe JF Lemieux P Krone W Gouni-Berthold I. The Whey Fermentation Product Malleable Protein Matrix Decreases Triglyceride Concentrations in Subjects With Hypercholesterolemia: A Randomized Placebo-Controlled Trial. J Dairy Sci (2011) 94:589–601. 10.3168/jds.2010-3115 21257028

[55] Heldenberg D Tamir I Levtow O Burstein Y Werbin B. Lipoprotein Measurements–a Necessity for Precise Assessment of Risk in Children From High-Risk Families. Arch Dis Childhood (1979) 54:695–8. 10.1136/adc.54.9.695 PMC1545826 518107

[56] Alzaabi A Al-Kaabi J Al-Maskari F Farhood AF Ahmed LA. Prevalence of Diabetes and Cardio-Metabolic Risk Factors in Young Men in the United Arab Emirates: A Cross-Sectional National Survey. Endocrinol Diabetes Metab (2019) 2:e00081. 10.1002/edm2.81 31592445 PMC6775448

[57] Nakagami T Nishimura R Sone H Tajima N. The Combination of Elevated Triglycerides and Abnormal Fasting Glucose Increases Risk of Cerebral Infarction in Patients With Mild to Moderate Hypercholesterolemia: A Post Hoc Analysis of the MEGA Study. J Cardiovasc Pharmacol Ther (2015) 20:169–73. 10.1177/1074248414537706 24906541

[58] Pursell N Gierut J Zhou W Dills M Diwanji R Gjorgjieva M. Inhibition of Glycogen Synthase II With RNAi Prevents Liver Injury in Mouse Models of Glycogen Storage Diseases. Mol Ther: J Am Soc Gene Ther (2018) 26:1771–82. 10.1016/j.ymthe.2018.04.023 PMC6035741 29784585

[59] Franco LM Krishnamurthy V Bali D Weinstein DA Arn P Clary B. Hepatocellular Carcinoma in Glycogen Storage Disease Type Ia: A Case Series. J Inherited Metab Dis (2005) 28:153–62. 10.1007/s10545-005-7500-2 15877204

[60] Calderaro J Labrune P Morcrette G Rebouissou S Franco D Prévot S. Molecular Characterization of Hepatocellular Adenomas Developed in Patients With Glycogen Storage Disease Type I. J Hepatol (2013) 58:350–7. 10.1016/j.jhep.2012.09.030 23046672

[61] Allende DS Gawrieh S Cummings OW Belt P Wilson L Van Natta M. Glycogenosis Is Common in Nonalcoholic Fatty Liver Disease and Is Independently Associated With Ballooning, But Lower Steatosis and Lower Fibrosis. Liver Int (2020) 41:996–1011. 10.1111/liv.14773 PMC8052274 33354866

[62] Ahmed-Sorour H Bailey CJ. Role of Ovarian Hormones in the Long-Term Control of Glucose Homeostasis, Glycogen Formation and Gluconeogenesis. Ann Nutr Metab (1981) 25:208–12. 10.1159/000176496 7305285

[63] Kuo T McQueen A Chen TC Wang JC. Regulation of Glucose Homeostasis by Glucocorticoids. Adv Exp Med Biol (2015) 872:99–126. 10.1007/978-1-4939-2895-8_5 26215992 PMC6185996

[64] Naiman S Huynh FK Gil R Glick Y Shahar Y Touitou N. SIRT6 Promotes Hepatic Beta-Oxidation *Via* Activation of Pparα. Cell Rep (2019) 29:4127–43.e8. 10.1016/j.celrep.2019.11.067 31851938 PMC7165364

[65] Orbe-Orihuela YC Lagunas-Martínez A Bahena-Román M Madrid-Marina V Torres-Poveda K Flores-Alfaro E. High Relative Abundance of Firmicutes and Increased TNF-α Levels Correlate With Obesity in Children. Salud Publica Mexico (2018) 60:5–11. 10.21149/8133 29689651

[66] Liu B Zhang Y Wang R An Y Gao W Bai L. Western Diet Feeding Influences Gut Microbiota Profiles in Apoe Knockout Mice. Lipids Health Dis (2018) 17:159. 10.1186/s12944-018-0811-8 30021609 PMC6052692

[67] Kehrmann J Effenberg L Wilk C Schoemer D Ngo Thi Phuong N Adamczyk A. Depletion of Foxp3(+) Regulatory T Cells is Accompanied by an Increase in the Relative Abundance of Firmicutes in the Murine Gut Microbiome. Immunology (2020) 159:344–53. 10.1111/imm.13158 PMC7011623 31755554

[68] Kameyama K Itoh K. Intestinal Colonization by a Lachnospiraceae Bacterium Contributes to the Development of Diabetes in Obese Mice. Microbes Environ (2014) 29:427–30. 10.1264/jsme2.ME14054 PMC4262368 25283478

[69] Vacca M Celano G Calabrese FM Portincasa P Gobbetti M De Angelis M. The Controversial Role of Human Gut Lachnospiraceae. Microorganisms (2020) 8:573. 10.3390/microorganisms8040573 PMC7232163 32326636

