# Supplementary figures and images for: Ovariectomy Impaired Hepatic Glucose and Lipid Homeostasis and Altered the Gut Microbiota in Mice With Different Diets
Source: Front Endocrinol (Lausanne). 2021 Jun 30;12:708838. doi: 10.3389/fendo.2021.708838 (PMC8278766; doi:10.3389/fendo.2021.708838)

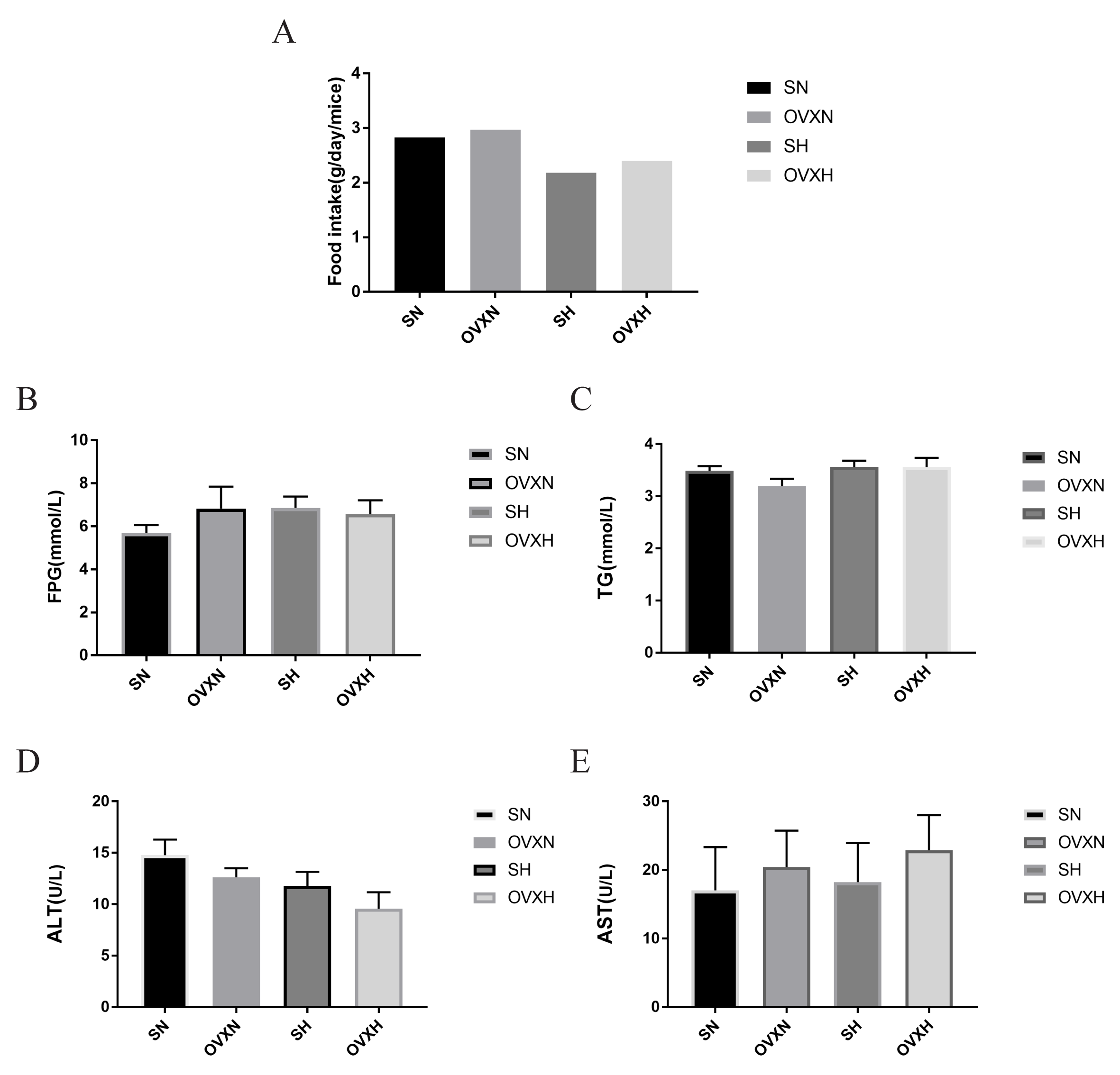

Supplement: Supplementary Figure 1 — The average food intake and the serum concentrations of glucose, TG, ALT and AST of mice with NFD or HFD. (A) The average daily food intake within 6weeks after operation of mice from the SN, OVXN, SH and OVXH groups. (B–E) The concentration of fasting plasm glucose (FPG), TG, ALT, and AST in the serum of mice from the SN, OVXN, SH and OVXH groups at six weeks after operation. SN, sham operated control mice fed with normal food diet; OVXN, ovariectomized mice fed with normal food diet; SH, sham operated control mice fed with high fat diet; OVXH, ovariectomized mice fed with high fat diet. [file Image_1.tif]

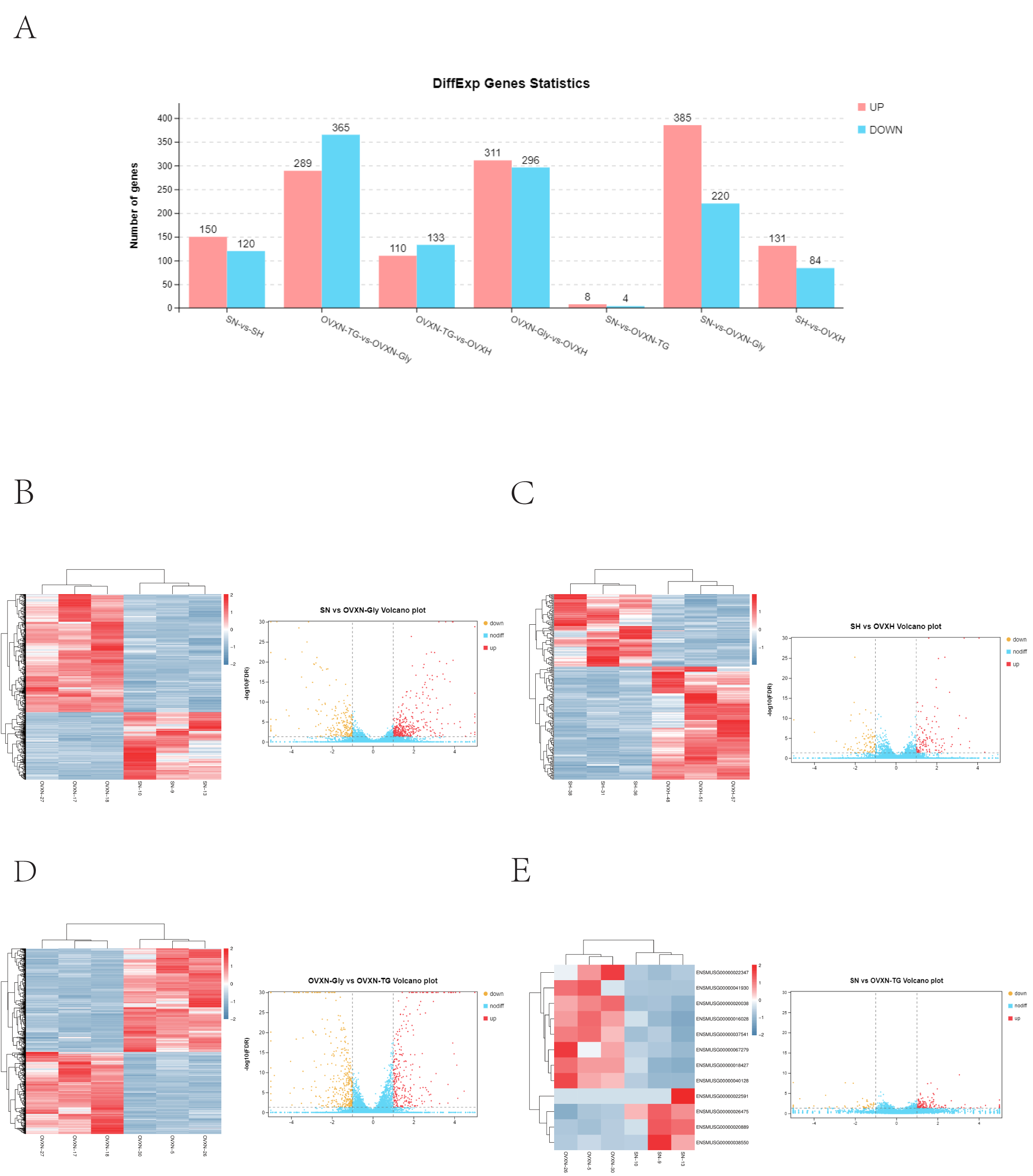

Supplement: Supplementary Figure 2 — Transcriptome analysis of the liver from ovariectomized and control mice with NFD or HFD. (A) Statistics of differentially expressed genes in the liver of mice compared between every group of SN, OVXN-Gly, OVXN-TG, SH and OVXH. (B) Heatmap and volcano pot showed differentially expressed genes in the liver of mice from the SN and OVXN-Gly groups. (C) Heatmap and volcano pot showed differentially expressed genes in the liver of mice from the SH and OVXH groups. (D) Heatmap and volcano pot showed differentially expressed genes in the liver of mice from the OVXN-Gly and OVXN-TG groups. (E) Heatmap and volcano pot showed differentially expressed genes in the liver of mice from the SN and OVXN-TG groups. SN, sham operated control mice fed with normal food diet; OVXN-Gly, ovariectomized mice fed with normal food diet and glycogen accumulated in its liver; OVXN-TG, ovariectomized mice fed with normal food diet and triglyceride accumulated in its liver; SH, sham operated control mice fed with high fat diet; OVXH, ovariectomized mice fed with high fat diet. [file Image_2.tif]

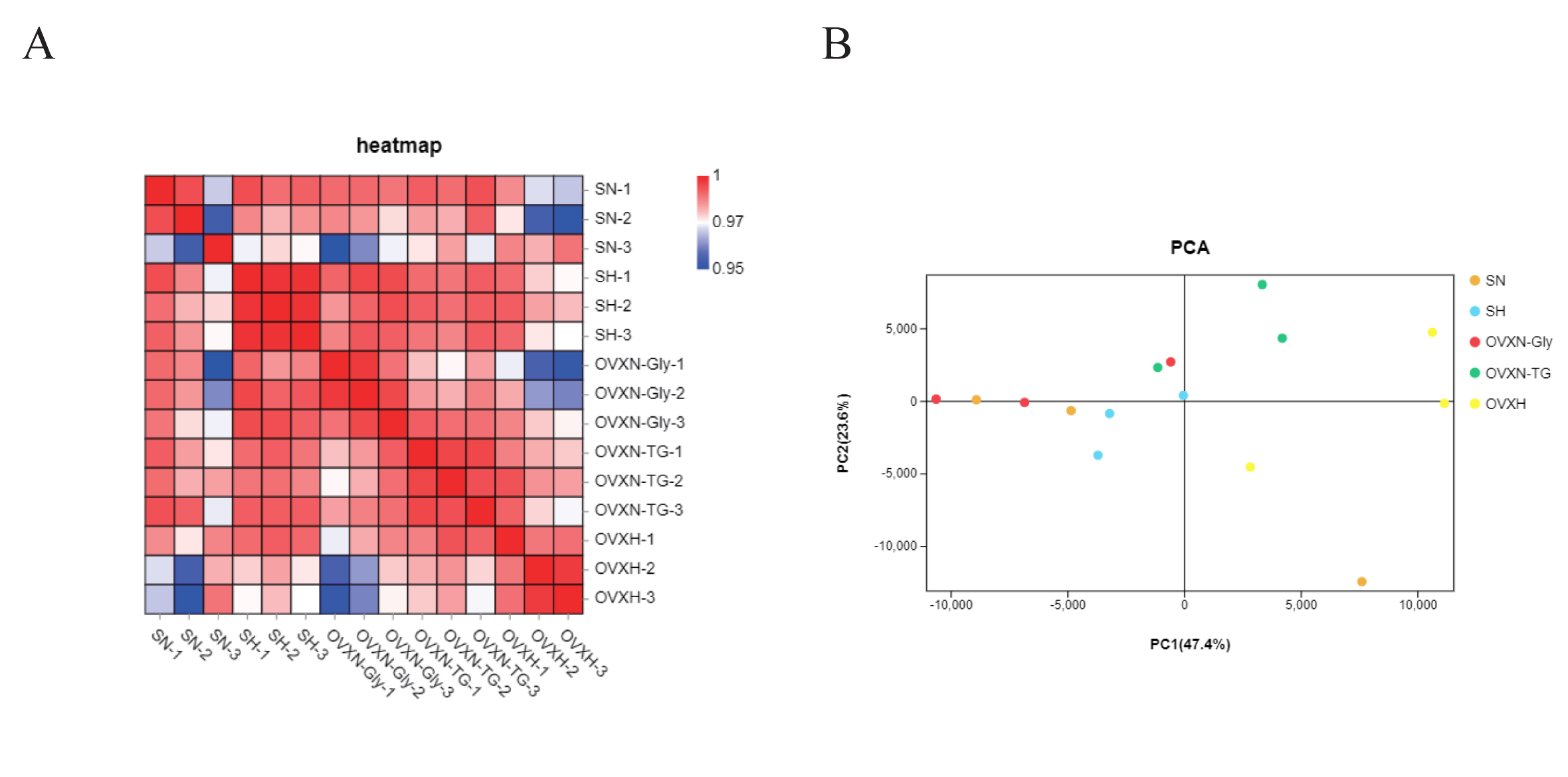

Supplement: Supplementary Figure 3 — Relationship analysis of liver tissues of the ovariectomized and control mice with NFD or HFD for transcriptional sequences. (A) Heatmap demonstrated the relationship of liver tissues of the SN, OVXN, SH and OVXH groups. (B) PCA analysis of the relationship of liver tissues of the SN, OVXN, SH and OVXH groups. SN, sham operated control mice fed with normal food diet; OVXN, ovariectomized mice fed with normal food diet; SH, sham operated control mice fed with high fat diet; OVXH, ovariectomized mice fed with high fat diet. [file Image_3.tif]

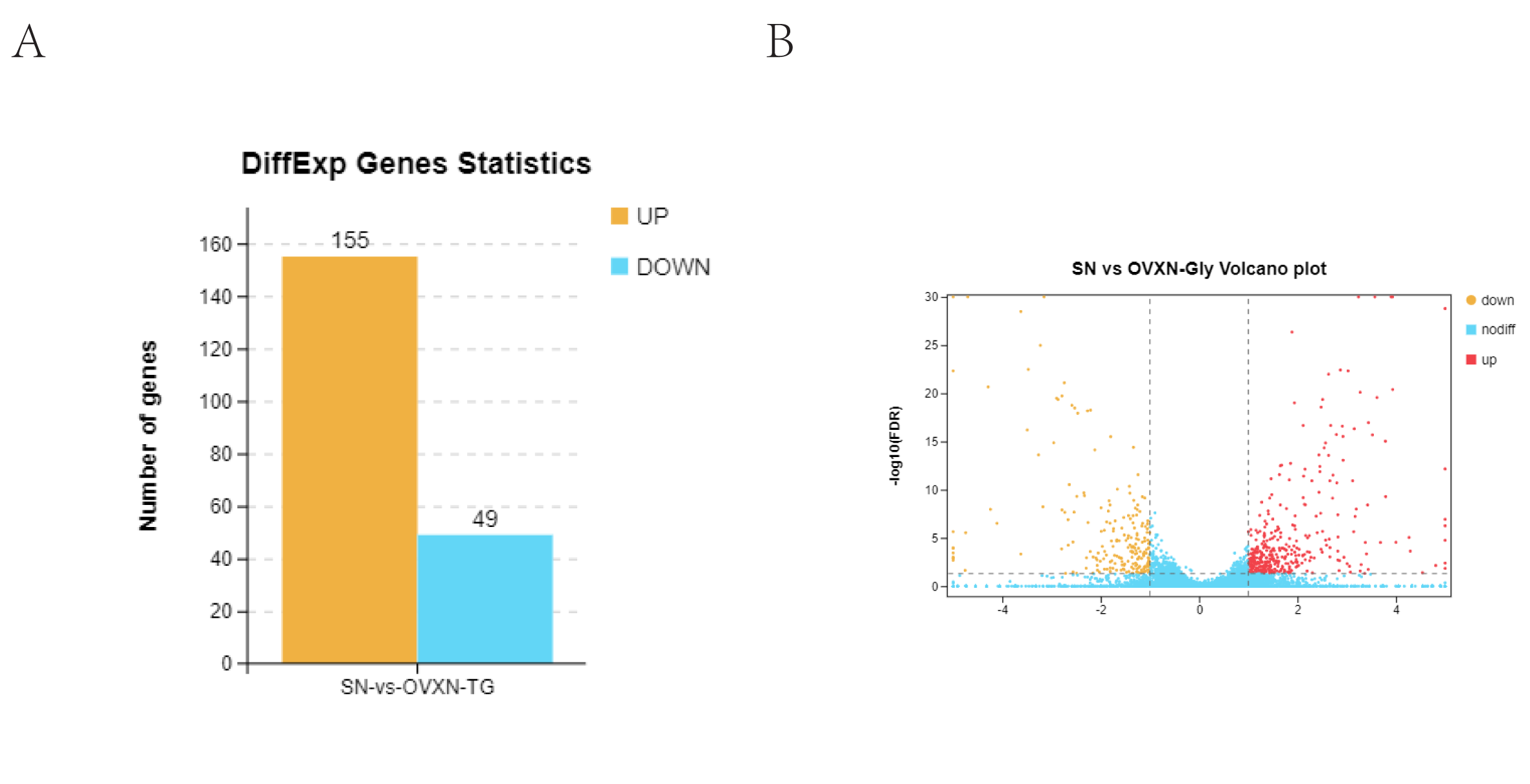

Supplement: Supplementary Figure 4 — Transcriptome analysis showing differentially expressed genes of the liver tissues from the SN and OVXN-TG mice. (A) Statistics of differentially expressed genes in the liver of the SN and OVXN-TG groups. (B) Volcano pot showed differentially expressed genes in liver of the SN and OVXN-TG groups. SN, sham operated control mice fed with normal food diet; OVXN-TG, ovariectomized mice fed with normal food diet and triglyceride accumulated in its liver. [file Image_4.tif]

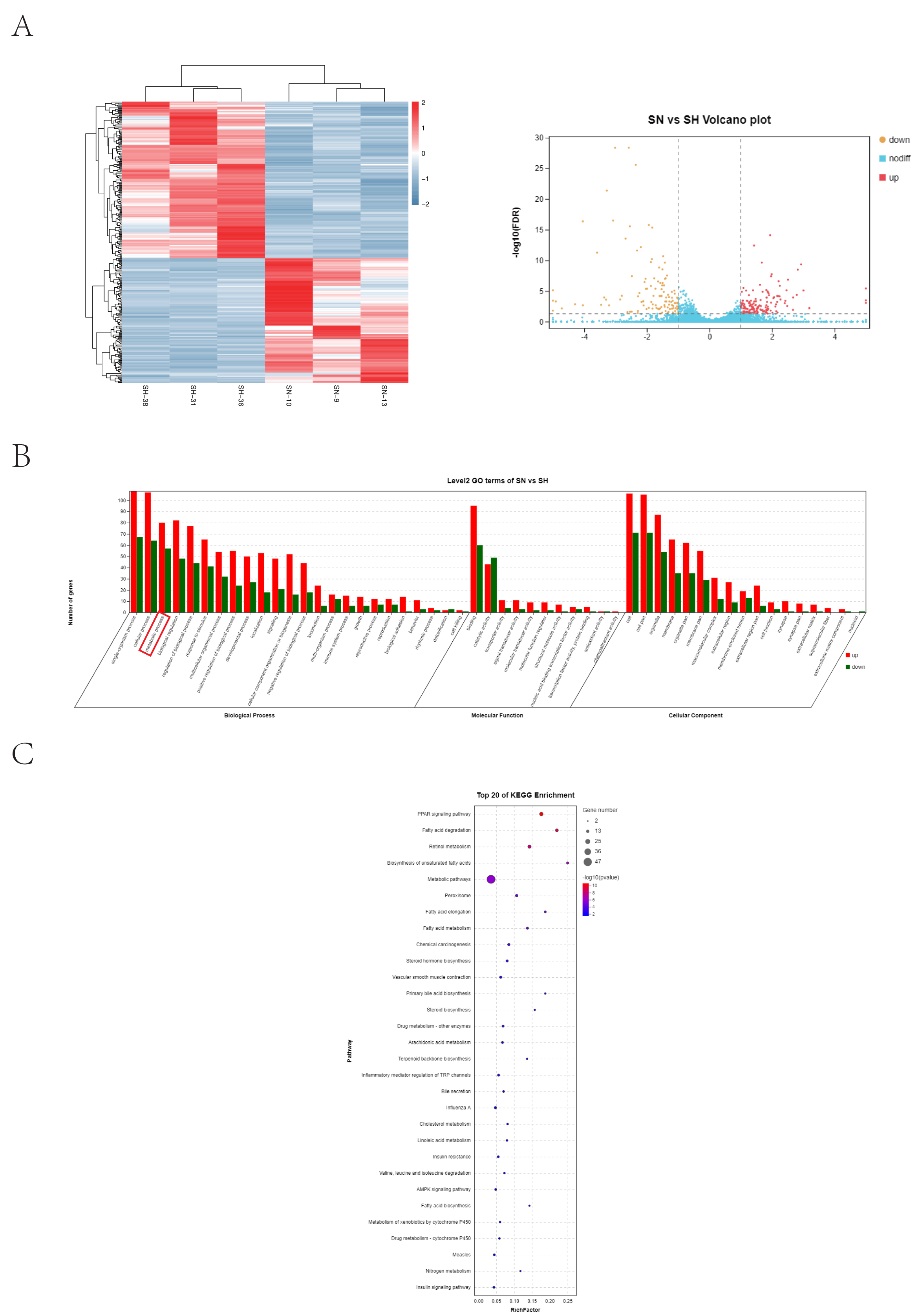

Supplement: Supplementary Figure 5 — Enriched molecular functions and biological pathways of the differentially expressed genes in the liver tissues of the SN and SH groups. (A) Heatmap and volcano pot showed differentially expressed genes in liver of the SN and SH groups. (B) GO classification of the source genes of the differentially expressed mRANs in the liver of the SN and SH groups. (C) KEGG assignments of the differentially expressed mRNAs in the liver of the SN and SH groups. SN, sham operated control mice fed with normal food diet; SH, sham operated control mice fed with high fat diet. [file Image_5.tif]

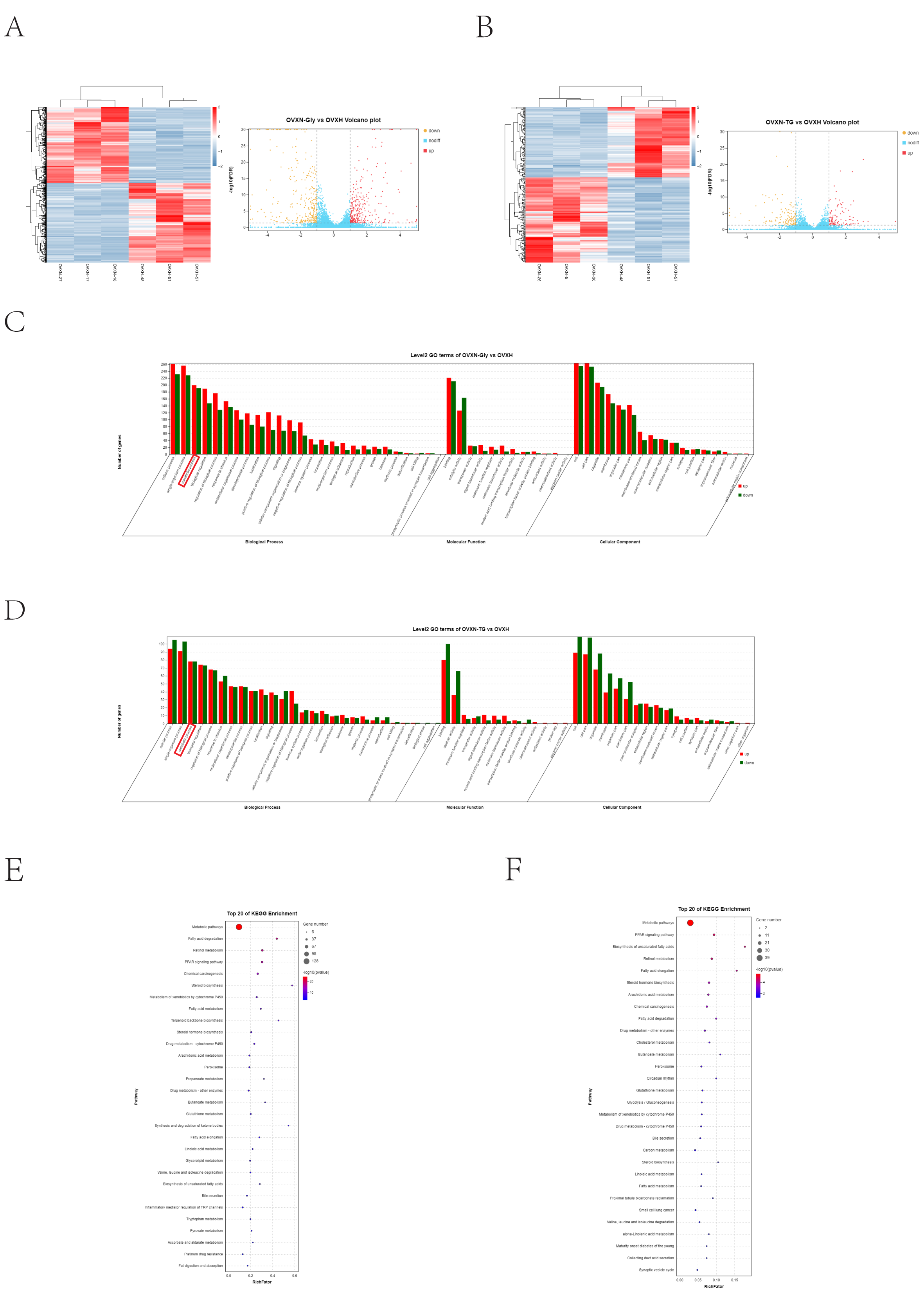

Supplement: Supplementary Figure 6 — Enriched molecular functions and biological pathways of the differentially expressed genes in the liver tissues of the OVXN and OVXH groups. (A) Heatmap and volcano pot showed differentially expressed genes in liver of the OVXN-Gly and OVXH groups. (B) Heatmap and volcano pot showed differentially expressed genes in liver of the OVXN-TG and OVXH groups. (C, D) GO classification of the source genes of the differentially expressed mRNAs in the liver of the OVXN-Gly vs. OVXH and OVXN-TG vs. OVXH respectively. (E, F) KEGG assignments of the differentially expressed mRNAs in the liver of the OVXN-Gly vs. OVXH and OVXN-TG vs. OVXH groups, respectively. OVXN-Gly, ovariectomized mice fed with normal food diet and glycogen accumulated in its liver; OVXN-TG, ovariectomized mice fed with normal food diet and triglyceride accumulated in its liver; OVXH, ovariectomized mice fed with high fat diet. [file Image_6.tif]

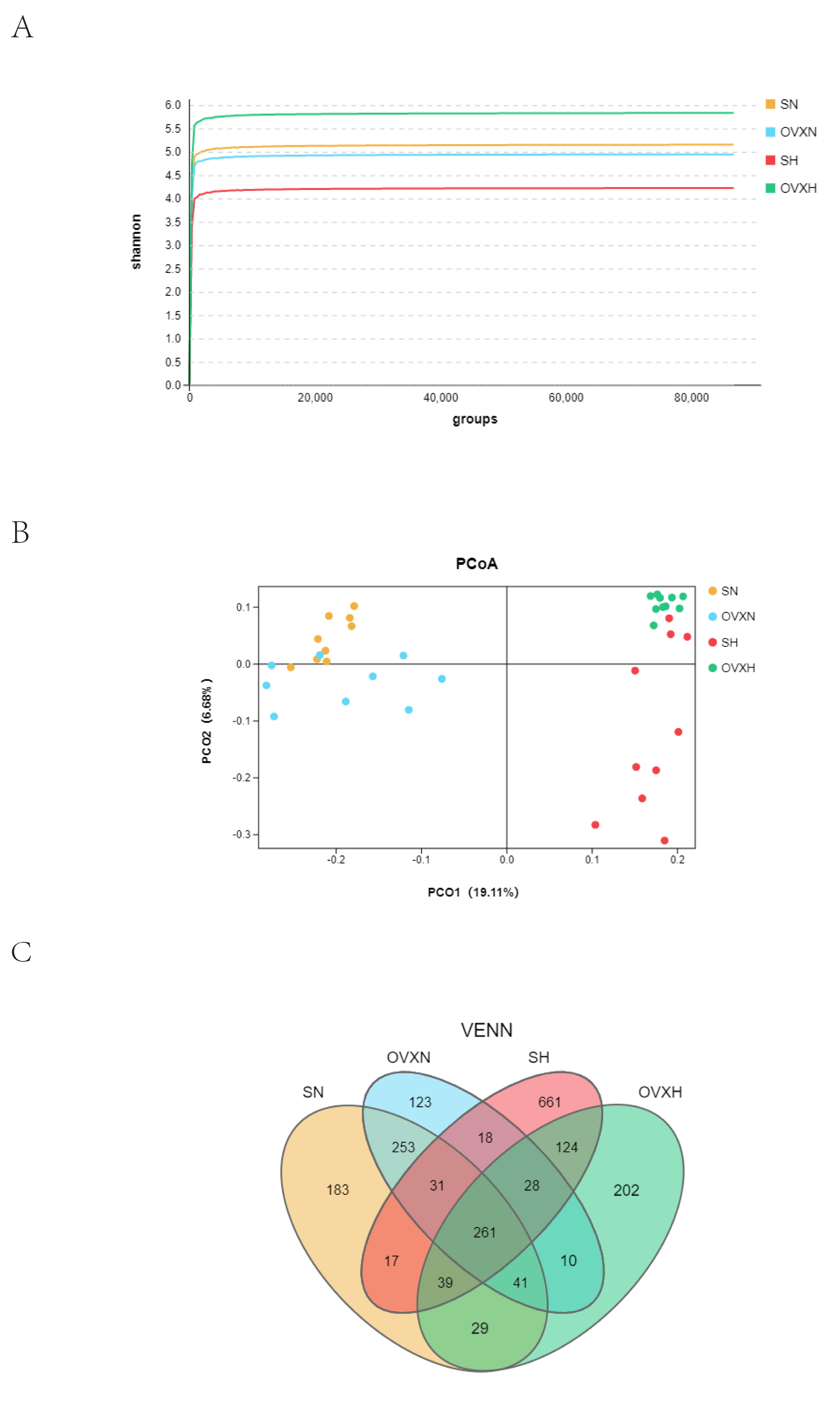

Supplement: Supplementary Figure 7 — Overview of 16S rDNA genes of the gut microbiota of the ovariectomized and control mice with NFD or HFD. (A) Shannon rarefaction curves for the SN, OVXN, SH, and OVXH groups. (B) PCoA analysis of the gut microbiota of the SN, OVXN, SH and OVXH groups. (C) Venn diagram showed the number of the OTUs of the SN, OVXN, SH and OVXH groups. SN, sham operated control mice fed with normal food diet; OVXN, ovariectomized mice fed with normal food diet; SH, sham operated control mice fed with high fat diet; OVXH, ovariectomized mice fed with high fat diet; OTU, operational taxonomic units; PCoA, principal co-ordinates analysis. [file Image_7.tif]

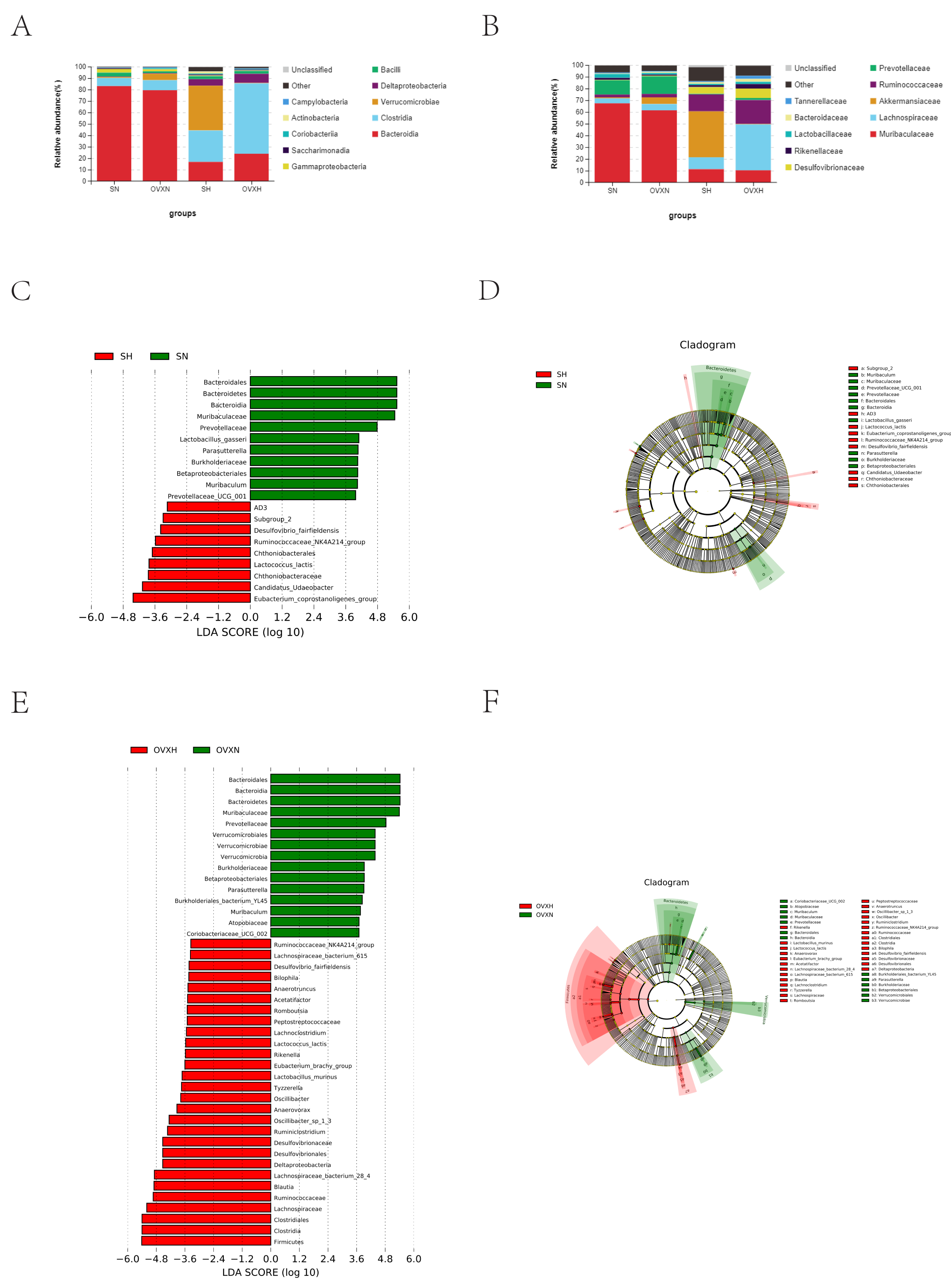

Supplement: Supplementary Figure 8 — Relative abundance of the gut microbiota of the SN, OVXN, SH and OVXH groups. (A, B) Relative abundance of gut microbiota at class and family levels of the SN, OVXN, SH and OVXH groups. Different colors were used to demonstrate each flora. (C, D) LDA scores and cladogram illustrated the predominant abundant taxons of the SN and SH groups. Red represented the bacteria distinctive for the SH group and green represented the bacteria distinctive for the SN group. (E, F) LDA scores and cladogram illustrated the predominant abundant taxons of the OVXN and OVXH groups. Red represented the bacteria distinctive for the OVXH group and green represented the bacteria distinctive for the OVXN group. SN, sham operated control mice fed with normal food diet; OVXN, ovariectomized mice fed with normal food diet; SH, sham operated control mice fed with high fat diet; OVXH, ovariectomized mice fed with high fat diet; LDA, Linear Discriminant Analysis. [file Image_8.tif]

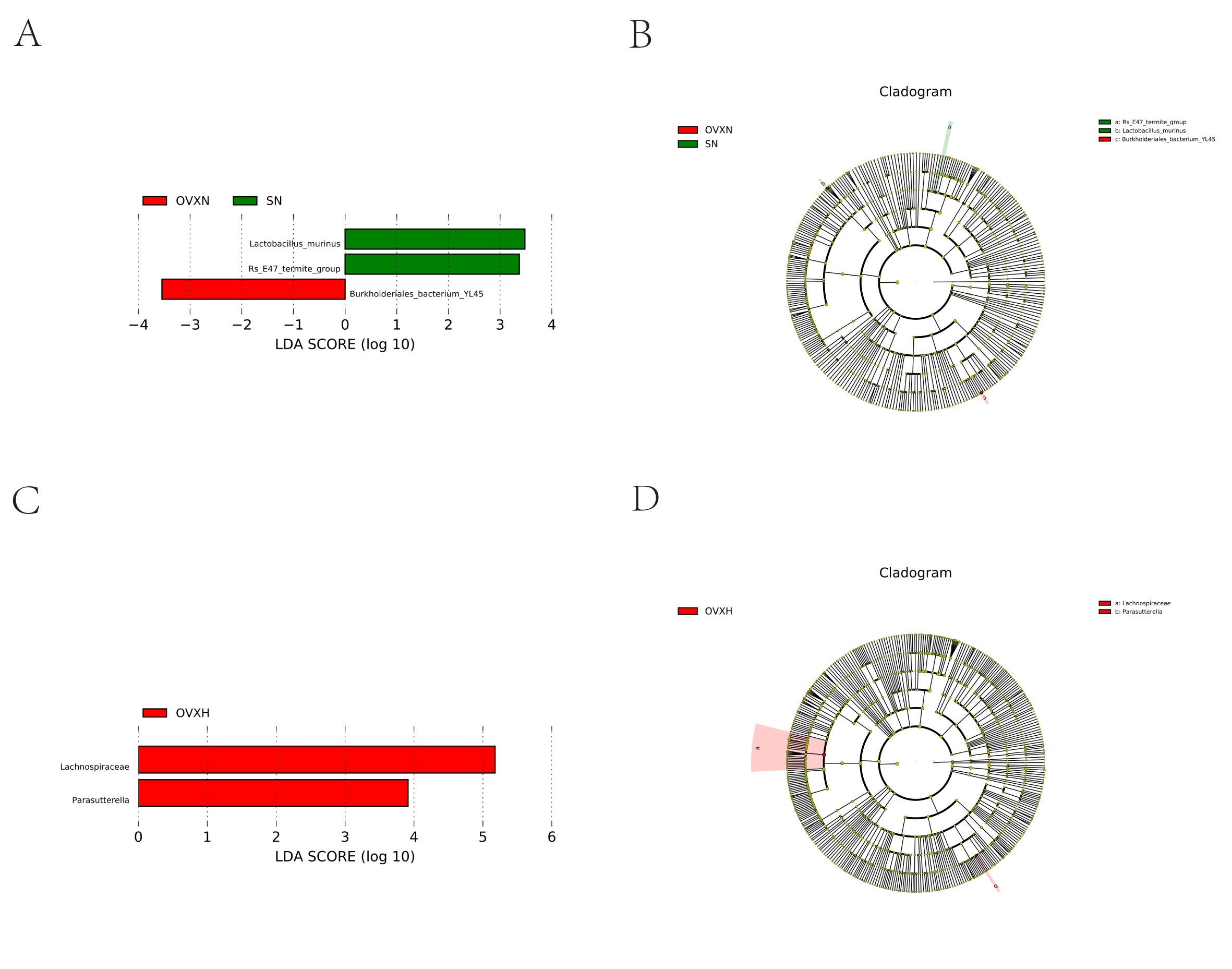

Supplement: Supplementary Figure 9 — LEFse analysis indicated bacterial taxa difference in abundance of gut microbiota of the ovariectomized and control mice with NFD or HFD. (A, B) LDA scores and cladogram illustrated the predominant abundant taxons of the SN and OVXN groups. Red represented the bacteria distinctive for the OVXN group and green represented the bacteria distinctive for the SN group. (C, D) LDA scores and cladogram illustrated the predominant abundant taxons of the SH and OVXH groups. Red represented the bacteria distinctive for the OVXH group and green represented the bacteria distinctive for the SH group. SN, sham operated control mice fed with normal food diet; OVXN, ovariectomized mice fed with normal food diet; SH, sham operated control mice fed with high fat diet; OVXH, ovariectomized mice fed with high fat diet; LDA, Linear Discriminant Analysis. [file Image_9.tif]

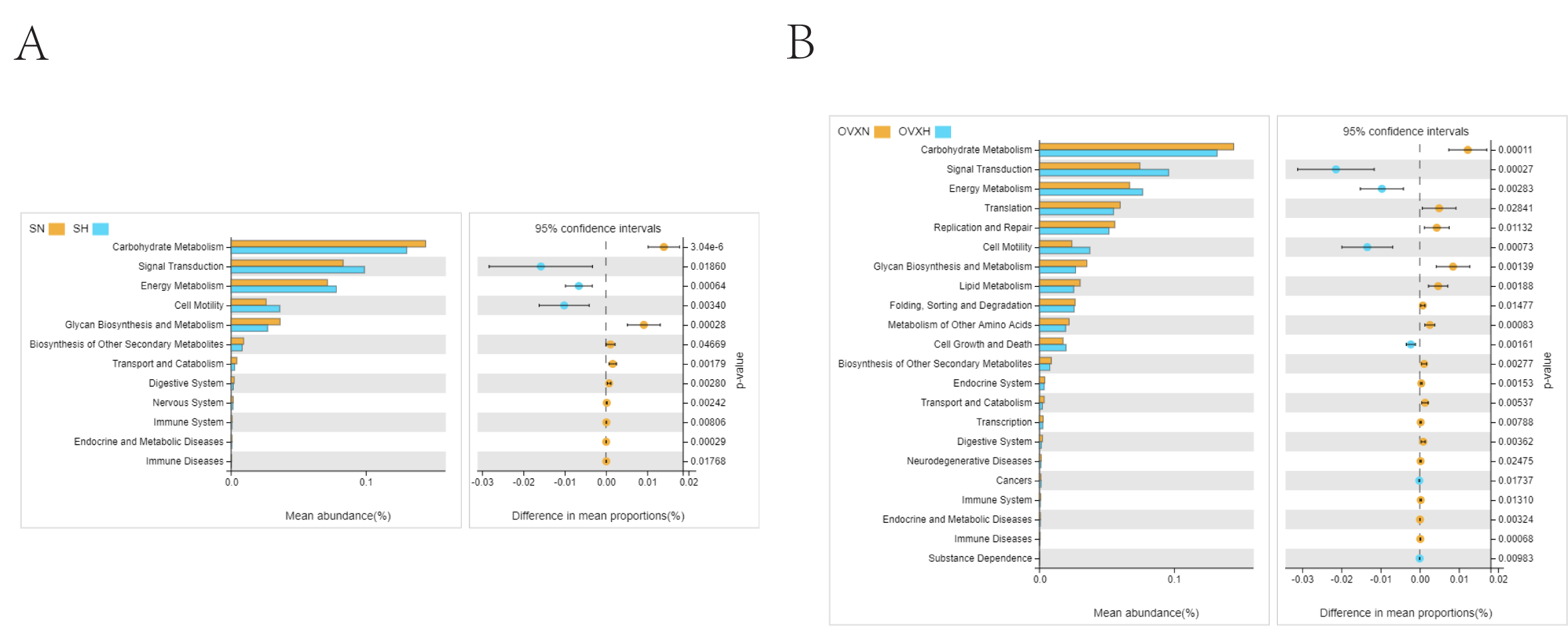

Supplement: Supplementary Figure 10 — KEGG enrichment analysis of the altered signaling pathways based on the relative abundance of the gut microbiota of the SN, OVXN, SH, OVXH groups. (A) Presentation of the altered KEGG pathways in the SN and SH groups. (B) Presentation of the altered KEGG pathways in the OVXN and OVXH groups (bar plots on the left side displayed the mean proportion of each KEGG pathway. Dot plots on the right side showed the differences in mean proportions between the two indicated groups using q-values). KEGG, Kyoto Encyclopedia of Genes and Genomes; SN, sham operated control mice fed with normal food diet; OVXN, ovariectomized mice fed with normal food diet; SH, sham operated control mice fed with high fat diet; OVXH, ovariectomized mice fed with high fat diet. [file Image_10.tif]
