# Supplementary material for: Ovariectomy Impaired Hepatic Glucose and Lipid Homeostasis and Altered the Gut Microbiota in Mice With Different Diets
Source: Front Endocrinol (Lausanne). 2021 Jun 30;12:708838. doi: 10.3389/fendo.2021.708838 (PMC8278766; doi:10.3389/fendo.2021.708838)
Supplement: Supplementary file 11 [file Table_1.docx]

Table S1. Summary of mice used in the present study

| Group of mice | SN | OVXN | SH | OVXH |
| --- | --- | --- | --- | --- |
| Numbers of mice | 12 | 11 | 13 | 13 |
| Histological staining | 8 | 8 | 8 | 8 |
| Transcriptome analysis | 3 | 3 for OVXN-Gly  3 for OVXN-TG | 3 | 3 |
| qRT-PCR | 8 | 3 for OVXN-Gly  (Repeat twice)  5 for OVXN-TG  (Repeat twice) | 8 | 8 |
| Western blot | 6 | 3 for OVXN-Gly  (Repeat twice)  5 for OVXN-TG  (One sample was repeated) | 6 | 6 |
| 16S rDNA gene analysis | 10 | 10 | 10 | 10 |
