# Supplementary material for: Ovariectomy Impaired Hepatic Glucose and Lipid Homeostasis and Altered the Gut Microbiota in Mice With Different Diets
Source: Front Endocrinol (Lausanne). 2021 Jun 30;12:708838. doi: 10.3389/fendo.2021.708838 (PMC8278766; doi:10.3389/fendo.2021.708838)
Supplement: Supplementary file 12 [file Table_2.docx]

Table S2. Primer sequences of genes for qPCR.

| Genes | Forward (from 5’to 3’) | Reverse (from 5’to 3’) |
| --- | --- | --- |
| GAPDH | GCATCCACTGGTGCTGCC | TCATCATACTTGGCAGGTTTC |
| Cyp3a41a | CAGAAAGGTAGCCCTACAGAGAG | ACGGGTCCCATATCGGTAGA |
| Perilipin2 | TGCTGTGTGGTGATCTGGAC | CTCTCATCACCACGCTCTGT |
| Fasn | CAAGTGCAAACCAGACTTCTAC | GCACTTTCTTTTCCGGTACTTT |
| ACLY | TTGGAGGCAGCATTGCAAAC | GCCAAAGACATGGATGGGGA |
| Acca1 | GGCCAGTGATATGCTGAGAT | AGGGTCAAGTGCTGCTCCA |
| Elovl5 | CTTCAGTTTGTGCTGACAATCA | AAGAGAGCAATCAGGGAAATCA |
| Elovl6 | TCACTCAGATGCTGATGGGC | GCCGATGTAGGCCTCAAAGA |
| FGF21 | CCTTGAAGCCAGGGGTCATT | AGGATCAAAGTGAGGCGATCC |
| GK | TCAGGGTTATATGCGCCTTATT | GAAACAAACAGCTTCTAGTGCA |
| SCD1 | AACATTCAATCCCGGGAGAATA | GAAACTTTCTTCCGGTCGTAAG |
| Ldlr | CAGAAGTCGACACTGTACTGAC | AAGATGGACAGGAACCTCATAC |
| DGAT1 | TGGTAGTGGGCCCAAGGTAG | TGCAGACGATGGCACCTCAG |
| GPAT1 | CAAATAGGCCTCTGGAGGAGC | GGCTTTGCTTACTGGTCCTGTATC |
| CD36 | CTTTGAAAGAACTCTTGTGGGG | GTCTGTGCCATTAATCATGTCG |
| Pnpla2 | CCTTAGGAGGAATGCCCTGC | CTCCAACAAGCGGATGGTGA |
| PGC1a | GGATATACTTTACGCAGGTCGA | CGTCTGAGTTGGTATCTAGGTC |
| Lpin2 | CGGTGCCGCAGTCTCAA | GAGCACTGGTAGGAGCCATC |
| Hepatic lipase | AAATGCAAGCTCAAAGCAGGG | CTTCTCCCAAAGGGCTGGTA |
| CHREBP | GATCTATCCGTGAACTTGCAGG | TGGATGACCTGTGATCGATG |
| Cyp4a31a | CCCTAAGGCTCTACCCACCT | AATGCAGTTCCTGGCTCCTC |
| PPARa | GAGCTGCAAGATTCAGAAGAAG | GAATCTTTCAGGTCGTGTTCAC |
| Acox1 | CCAATGCTGGTATCGAAGAATG | CGACTGAACCTGGTCATAGATT |
| Acadm | TCAGAGTGCCTAAGGAAAATGT | CGACTGTAGGTCTGGTTCTATC |
| Cyp4a10 | GACCCTAGACACTGTCATGAAA | AAAGATATTCCTCACACGGGAG |
| Cyp4a14 | GTTGAATGGGAAGAAGTGGTTC | GCTGAAAGCACACTTCATAACA |
| HMGCR | GGTGCAAAGTTCCTTAGTGATG | GAATAGACACACCACGTTCATG |
| SREBP1 | GCTACCGGTCTTCTATCAATGA | CGCAAGACAGCAGATTTATTCA |
| SREBP2 | GGCTGTCGGGTGTCATGG | ACAAACTGTAGCATCTCGTCGAT |
| Cyp7a1 | GTGATGTTTGAAGCCGGATATC | TTTATGTGCGGTCTTGAACAAG |
| LXRa | AAAGAGCCTCCAGGGACAGTG | GATGCAAGTGTTGCCTCCCT |
| LXRβ | TTTCCAGGGCAACAGAGTCG | TCACGAAGCAGCCTTGTTCC |
| PPARγ | CCAAGAATACCAAAGTGCGATC | TCACAAGCATGAACTCCATAGT |
| MVK | CTGAAGTCAATCAACAAGTGGG | CTCTTCAAGGAAGACATCGTCC |
| PYGL | ATGTGAAGCGGATCCACGAG | TGATATCCTGGGGCAGCTTTG |
| G6PC | GCTGGAGTCTTGTCAGGCATT | AGAATCCAAGCGCGAAACCA |
| GYS2 | ATCACCACCAACGACGGA | GCCTCCTCTTCCTCATCATACC |
