# Supplementary material for: Ovariectomy Impaired Hepatic Glucose and Lipid Homeostasis and Altered the Gut Microbiota in Mice With Different Diets
Source: Front Endocrinol (Lausanne). 2021 Jun 30;12:708838. doi: 10.3389/fendo.2021.708838 (PMC8278766; doi:10.3389/fendo.2021.708838)
Supplement: Supplementary file 21 [file Table_11.docx]

Table S11. The comparison of the fold-change in RNA-seq and qPCR validation analysis of the present study

| Gene |  | SN vs OVXN-Gly | SN vs OVXN-TG | OVXN-Gly vs OVXN-TG | SH vs OVXH |
| --- | --- | --- | --- | --- | --- |
| Cyp3a41a | RNAseq | 0.169072279 | 0.953503746 | 5.639622027 | 0.21885491 |
|  | qPCR | 0.035358168 | 0.09082636 | 2.568751873 | 0.0714455 |
| Cyp4a31 | RNAseq | 0.106850608 | 1.032679396 | 9.664702994 | 2.65843842 |
|  | qPCR | 0.075976864 | 0.386731858 | 5.090126608 | 0.32777534 |
| Cyp4a10 | RNAseq | 0.144779144 | 1.036217984 | 7.15723245 | 2.25272277 |
|  | qPCR | 0.154719409 | 1.227160567 | 7.931523083 | 0.47783662 |
| Cyp4a14 | RNAseq | 0.081104992 | 0.992419563 | 12.23623286 | 2.51103227 |
|  | qPCR | 0.078107742 | 1.088941477 | 13.94153061 | 0.54400847 |
| Fasn | RNAseq | 11.88999467 | 1.371245779 | 0.115327703 | 0.66603025 |
|  | qPCR | 6.9128503 | 1.03692115 | 0.149999075 | 0.85505800 |
| perilipin 2 | RNAseq | 0.28489326 | 1.036584313 | 3.638500657 | 2.39983554 |
|  | qPCR | 0.21790881 | 1.36814108 | 6.278502817 | 0.91774105 |
| Acly | RNAseq | 9.450919121 | 1.281066176 | 0.135549375 | 0.77038512 |
|  | qPCR | 7.278292156 | 1.33437174 | 0.183335831 | 1.33047093 |
| Acca1 | RNAseq | 2.398811062 | 1.119152236 | 0.466544553 | 1.03374447 |
|  | qPCR | 3.116840853 | 1.187208911 | 0.380901357 | 3.20528929 |
| Elovl5 | RNAseq | 0.582584424 | 1.438430921 | 2.469051455 | 1.46774279 |
|  | qPCR | 0.598880712 | 1.63592566 | 2.731638585 | 1.02777150 |
| Elovl6 | RNAseq | 5.825822166 | 1.563946407 | 0.268450763 | 0.78064013 |
|  | qPCR | 9.08291691 | 1.148082632 | 0.126400213 | 0.90704290 |
| FGF21 | RNAseq | 0.019523624 | 1.342444358 | 68.76000007 | 16.9058824 |
|  | qPCR | 0.112170402 | 2.058456424 | 18.35115499 | 1.19411363 |
| GK | RNAseq | 0.458140004 | 1.330441799 | 2.904007045 | 1.58808864 |
|  | qPCR | 0.541582661 | 1.680864798 | 3.103616344 | 1.05428653 |
| SCD1 | RNAseq | 2.328899559 | 0.939339865 | 0.403340651 | 0.38100904 |
|  | qPCR | 1.584336632 | 1.146549331 | 0.723677852 | 0.27797026 |
| LDLR | RNAseq | 2.803846154 | 1.488653846 | 0.530932785 | 0.6868327 |
|  | qPCR | 2.221544056 | 0.970294681 | 0.436765896 | 0.49922832 |
| DGAT1 | RNAseq | 0.591492777 | 0.901284109 | 1.523744912 | 1.18778046 |
|  | qPCR | 0.428263431 | 0.471941841 | 1.101989585 | 0.80788277 |
| CD36 | RNAseq | 0.525105868 | 1.221886133 | 2.326932924 | 3.49351404 |
|  | qPCR | 0.483428275 | 1.027218495 | 2.124862257 | 0.90516400 |
| Pnpla2 | RNAseq | 0.284963849 | 0.697842563 | 2.448881028 | 0.91813541 |
|  | qPCR | 0.403809207 | 0.948058596 | 2.347788457 | 0.82633719 |
| PGC1a | RNAseq | 1.040389972 | 1.193593315 | 1.147255689 | 0.54865182 |
|  | qPCR | 2.217484125 | 0.986044428 | 0.444668089 | 0.95918155 |
| Lpin2 | RNAseq | 0.57686822 | 0.733803 | 1.272046153 | 0.9755639 |
|  | qPCR | 0.836348776 | 1.030119698 | 1.231686741 | 1.15650246 |
| Hepatic lipase | RNAseq | 1.506078953 | 1.087837173 | 0.722297573 | 1.11327675 |
|  | qPCR | 1.592857656 | 1.203743215 | 0.755712986 | 0.72607919 |
| CHREBP | RNAseq | 3.197179044 | 1.6318365 | 0.510398848 | 1.41223500 |
|  | qPCR | 3.255460106 | 1.122243717 | 0.344726607 | 1.13378558 |
| PPARa | RNAseq | 0.478073881 | 1.25627832 | 2.627791165 | 1.5759352 |
|  | qPCR | 0.643585889 | 1.203770203 | 1.87041112 | 1.31854184 |
| ACOX1 | RNAseq | 0.407066153 | 1.424750205 | 3.500045867 | 1.44015476 |
|  | qPCR | 0.412878985 | 1.164463165 | 2.820349807 | 0.91633394 |
| Acadm | RNAseq | 0.423039937 | 1.043047751 | 2.465601137 | 1.57659710 |
|  | qPCR | 0.497844565 | 1.438834863 | 2.890128693 | 1.55849126 |
| HMGCR | RNAseq | 6.956123824 | 0.886675639 | 0.127466914 | 0.33062510 |
|  | qPCR | 5.416469459 | 0.803747152 | 0.148389492 | 0.4628119 |
| SREBP1 | RNAseq | 9.000000002 | 1.368920033 | 0.152102226 | 0.89431515 |
|  | qPCR | 7.852155727 | 1.382620849 | 0.17608169 | 1.0306897 |
| SREBP2 | RNAseq | 2.349819331 | 1.25564589 | 0.534358481 | 0.61506822 |
|  | qPCR | 2.311816396 | 1.544389814 | 0.668041725 | 1.00771509 |
| Cyp7a1 | RNAseq | 0.849278281 | 1.491104397 | 1.755731225 | 0.24832215 |
|  | qPCR | 1.14650403 | 1.72626992 | 1.505681511 | 0.23914865 |
| LXRα | RNAseq | 1.100137615 | 0.981788991 | 0.8924238 | 1.0493730 |
|  | qPCR | 1.192941832 | 1.32889969 | 1.113968556 | 1.20196168 |
| LXRβ | RNAseq | 0.832727933 | 1.103886669 | 1.325627045 | 1.29926249 |
|  | qPCR | 1.004469435 | 1.106014732 | 1.101093466 | 0.81790773 |
| PPARγ | RNAseq | 0.862385321 | 0.981651376 | 1.138297872 | 2.1637255 |
|  | qPCR | 0.878544021 | 1.197407828 | 1.362945738 | 1.27450089 |
| PYGL | RNAseq | 2.645186235 | 1.367616102 | 0.517020724 | 0.88173546 |
|  | qPCR | 3.009031156 | 1.931766614 | 0.641989569 | 0.94551316 |
| G6PC | RNAseq | 1.828606422 | 3.060167643 | 1.673497153 | 1.26185791 |
|  | qPCR | 2.857802269 | 3.138863546 | 1.098348748 | 1.17008099 |
| GYS2 | RNAseq | 0.456592035 | 0.590110925 | 1.292424922 | 1.12657037 |
|  | qPCR | 1.50206081 | 0.725240559 | 0.482830358 | 1.26522823 |
